# Molecular characterization of *capulet2* reveals the importance of *ANAPHASE PROMOTING COMPLEX 6* maternal expression in endosperm development

**DOI:** 10.1093/plphys/kiag647

**Published:** 2026-09-01

**Authors:** Yuri S van Ekelenburg, Ida V Myking, Cathal Meehan, Morten P Hauger, Shinichiro Komaki, Keiko Sugimoto, José Gutierrez-Marcos, Paul E Grini

**Affiliations:** Section for Genetics and Evolutionary Biology, Department of Biosciences, University of Oslo, Oslo 0316, Norway; Section for Genetics and Evolutionary Biology, Department of Biosciences, University of Oslo, Oslo 0316, Norway; School of Life Science, University of Warwick, Coventry CV4 7AL, United Kingdom; Section for Genetics and Evolutionary Biology, Department of Biosciences, University of Oslo, Oslo 0316, Norway; Nara Institute of Science and Technology, Division of Biological Science, 8916-5 Takayama, Ikoma, Nara 630-0192, Japan; School of Biological Science and Technology, College of Science and Engineering, Kanazawa University, Kakuma-machi, Kanazawa 920-1192, Japan; RIKEN, Center for Sustainable Resource Science, Yokohama, Kanagawa 230-0045, Japan; School of Life Science, University of Warwick, Coventry CV4 7AL, United Kingdom; Section for Genetics and Evolutionary Biology, Department of Biosciences, University of Oslo, Oslo 0316, Norway

## Abstract

Flowering plants are characterized by a double fertilization event, and the fertilized female gametes develop into the endosperm and embryo. Genomic imprinting promotes parental allele-specific gene expression in the endosperm by epigenetic modifications such as DNA methylation. Similarly, gametophyte maternal effects influence gene function in the female gametophyte that affects development of the endosperm and embryo postfertilization. While many imprinted genes do not display a seed phenotype upon mutation, gametophyte maternal effect mutants are characterized by distorted seed development upon maternal transmission of the mutant allele. Here, we have investigated the gametophyte maternal effect mutant *capulet2* (*cap2*). We have established *CAP2* to be encoded by *ANAPHASE PROMOTING COMPLEX 6* (*APC6*), a subunit of the anaphase promoting complex/cyclosome (APC/C). Investigation of further *cap2/apc6* alleles revealed female gametophyte maternal effects both in mutant segregation and seed phenotype, and both *cap2* and *apc6* phenotypes were rescued by an *APC6* transgene. Furthermore, we have demonstrated that *APC6* is a maternally expressed imprinted gene, in line with the observed female gametophyte maternal effect phenotype. To this end, we observed irregular nuclear division of the endosperm coenocyte in *cap2/apc6* mutants, suggesting a role for the APC/C in early endosperm development. We have further demonstrated that similar endosperm defects are also produced by mutation of *APC1*, another subunit of the APC/C. Similar to *CAP2/APC6, APC1* is imprinted and only expressed from the maternal allele, suggesting a maternal bias in the control of APC/C in the developing endosperm.

## Introduction

Seed development in angiosperms is initiated by a double fertilization event forming the diploid embryo and triploid endosperm (Nowack et al. 2010). The embryo develops into the next generation and receives nutrients from the mother plant through the endosperm. Due to the triploid nature of the endosperm, a balanced parental gene expression is important (Barton et al. 1984; Birchler 1993). While most genes are expressed equally from both parental alleles (biparentally expressed genes [BEGs]), some genes have been found to be regulated in a parent-of-origin dependent manner (Köhler and Grossniklaus 2005; Nowack et al. 2010). Parent-of-origin specific gene expression, also called genomic imprinting, has been described in mammals, filamentous fungi, and flowering plants (Martienssen and Colot 2001; Feil and Berger 2007). In genomic imprinting, an epigenetic mark is established in the gametes, resulting in silencing of the allele postfertilization (Montgomery and Berger 2021).

Currently, 2 major epigenetic regulatory mechanisms are known to be involved in the establishment of imprinting, DNA methylation, and histone modification. Maternally expressed genes (MEGs) are mainly hypothesized to be regulated by DNA methylation and removal of methylated cytosines by DNA glycosylases. Several DNA methyltransferases have been identified in *Arabidopsis thaliana* (*A. thaliana*), including DNA METHYLTRANSFERASE 1 (MET1) (Finnegan and Dennis, 1993), CHROMOMETHYLASE 3 (CMT3) (Lindroth et al. 2001) and DOMAINS REARRANGED METHYLASE 1 & 2 (DRM1/DRM2) (Cao and Jacobsen 2002). It has been shown that DNA methylation marks established by MET1 (Xiao et al. 2003; Kinoshita et al. 2004; Gehring et al. 2006; Shirzadi et al. 2011), DRM1/DRM2 (Matzke and Mosher 2014; Hornslien et al. 2019) and CMT3, guided by histone H3 lysine nine di-methylation (H3K9me2) (Stroud et al. 2014; Moreno-Romero et al. 2019), can regulate the imprinting state of specific genes. In addition, RNA-directed de novo DNA methylation (RdDM) guided by small RNAs has been demonstrated (Vu et al. 2013; Hornslien et al. 2019; Satyaki and Gehring 2019; Batista and Köhler 2020).

Paternally expressed genes (PEGs) are mainly considered to be regulated by silencing of the maternal allele in the central cell by the FERTILIZATION INDEPENDENT SEED 2-Polycomb Repressive Complex 2 (FIS-PRC2) complex (Gehring 2013). Upon recruitment of FIS-PRC2 to DNA hypomethylated loci on the maternal allele, the histone methyltransferase subunit MEDEA (MEA) trimethylates histone H3 lysine 27 (H3K27me3), a mark for gene repression (Rodrigues and Zilberman 2015; Moreno-Romero et al. 2016). Several genes have been shown to exhibit maternal silencing by the FIS-PRC2 complex, including *PHERES1* (*PHE1*), and display interaction between DNA and histone methylation (Köhler et al. 2005; Makarevich et al. 2008; Hornslien et al. 2019; Batista and Köhler 2020).

Similar to imprinted genes, gametophyte parental effect genes affect postfertilization seed development (St Johnston and Nüsslein-Volhard, 1992; Colombo et al. 1997). Although the outcomes of parental effect genes and imprinted genes are relatively similar, the genetic phenomena are distinct and have different molecular origins (Wolf and Wade 2009). The epigenetic marks for imprinted genes are established prior to fertilization and affect gene expression postfertilization (Montgomery and Berger 2021), whereas parental effects are caused by prefertilization products (ie mRNA and proteins) that become active and execute their effect postfertilization, irrespective of gene expression (St Johnston and Nüsslein-Volhard, 1992; Colombo et al. 1997).

Gametophyte maternal effect mutants have been characterized by normal gametophyte development, followed by disrupted embryo and/or endosperm development when the mutant allele is transmitted through the female parent (Yadegari and Drews 2004). Gametophyte maternal effect mutants have been identified in both *Arabidopsis* and maize (Evans and Kermicle 2001; Grini et al. 2002; Olsen 2004; Pagnussat et al. 2005; Gutiérrez-Marcos et al. 2006; Phillips and Evans 2011; Chettoor et al. 2016), including the *capulet* (*cap*) mutants (*cap1* and *cap2*), which show developmental arrest in the early developing embryo and endosperm (Grini et al. 2002). The *cap2* allele is only lethal when transmitted maternally, indicative of a gametophytic maternal effect mutant. Gametophyte maternal effects could be explained by maternally deposited RNA or protein or by genomic imprinting. However, it remains technologically challenging to identify the origin of transcripts in the endosperm directly after fertilization, ie whether transcripts come from the female central cell as maternal carryover or if they have been transcribed de novo (Evans and Kermicle 2001).

In this study, we have identified the molecular nature of the gametophytic maternal effect mutant *cap2.* Using whole genome sequencing and single nucleotide polymorphism (SNP) analysis, we identified the causative *cap2* SNP to be located in an intron splice donor site of the *ANAPHASE PROMOTING COMPLEX 6* (*APC6*), a subunit of the ANAPHASE PROMOTING COMPLEX/CYCLOSOME (APC/C). Two independent T-DNA insertion lines, *apc6-2* and *apc6-3*, were thoroughly analyzed and phenocopied *cap2,* both in terms of seed phenotypes and frequency. Moreover, *cap2*, *apc6-2*, and *apc6-3* could be rescued by molecular complementation using an *APC6* transgene. In line with the gametophyte maternal effect phenotype, *APC6* was shown to be a maternally expressed imprinted gene. Last, we demonstrated that mutation of *APC1*, another subunit of the APC/C, displays a female gametophyte phenotype and enhances the frequency of disrupted endosperm development in an *apc6* genetic background. Similar to *cap2/apc6, APC1* is imprinted and expressed only from the maternal allele, suggesting a maternal bias to the anaphase promoting complex (APC/C).

## Results

### *Capulet2* causative mutation is located in *APC6*

The *capulet2* mutant was identified in an ethyl methanesulfonate (EMS)-induced gametophytic mutant screen by a segregation distortion assay using the *multiply marked chromosome 1* (*mm1*) line (Grini et al. 1999). The location of *cap2* was determined between the flanking genetic markers *ap1* and *gl2* (Fig. 1a) at a genome interval between *ADH1* and *gl2* using molecular markers (Grini et al. 2002). To determine the causative mutation of *cap2*, we employed whole genome sequencing. For this purpose, gDNA was isolated from 12 *cap2/CAP2* (*cap2/+*) and 12 *CAP2/CAP2* (+/+) plants. We performed a nucleotide polymorphism analysis between pairs of *cap2/*+ and *CAP2/CAP2* individuals, which revealed a clear linkage break on chromosome 1 (Figure S1). We found 100 to 180 candidate SNPs in the *ADH1* and *gl2* interval (Fig. 1b, Data S1). Candidate SNPs were further selected by their base alignment quality score (BAQ) of ≥70 (Li, 2011) and the presence of guanine to adenine conversion as the mutation arose in a population treated with ethyl methanesulfonate (EMS) with an allele frequency of 0.25 to 0.75 (Table S1a) and conserved across all 12 comparisons (Table S1b). We found 3 SNPs that met these requirements—1 in the *ANAPHASE PROMOTING COMPLEX 6* (*APC6*), 1 in the *STRUBBELIG RECEPTOR FAMILY 5* (*SRF5*), and 1 in an intergenic region.

**Figure 1:**
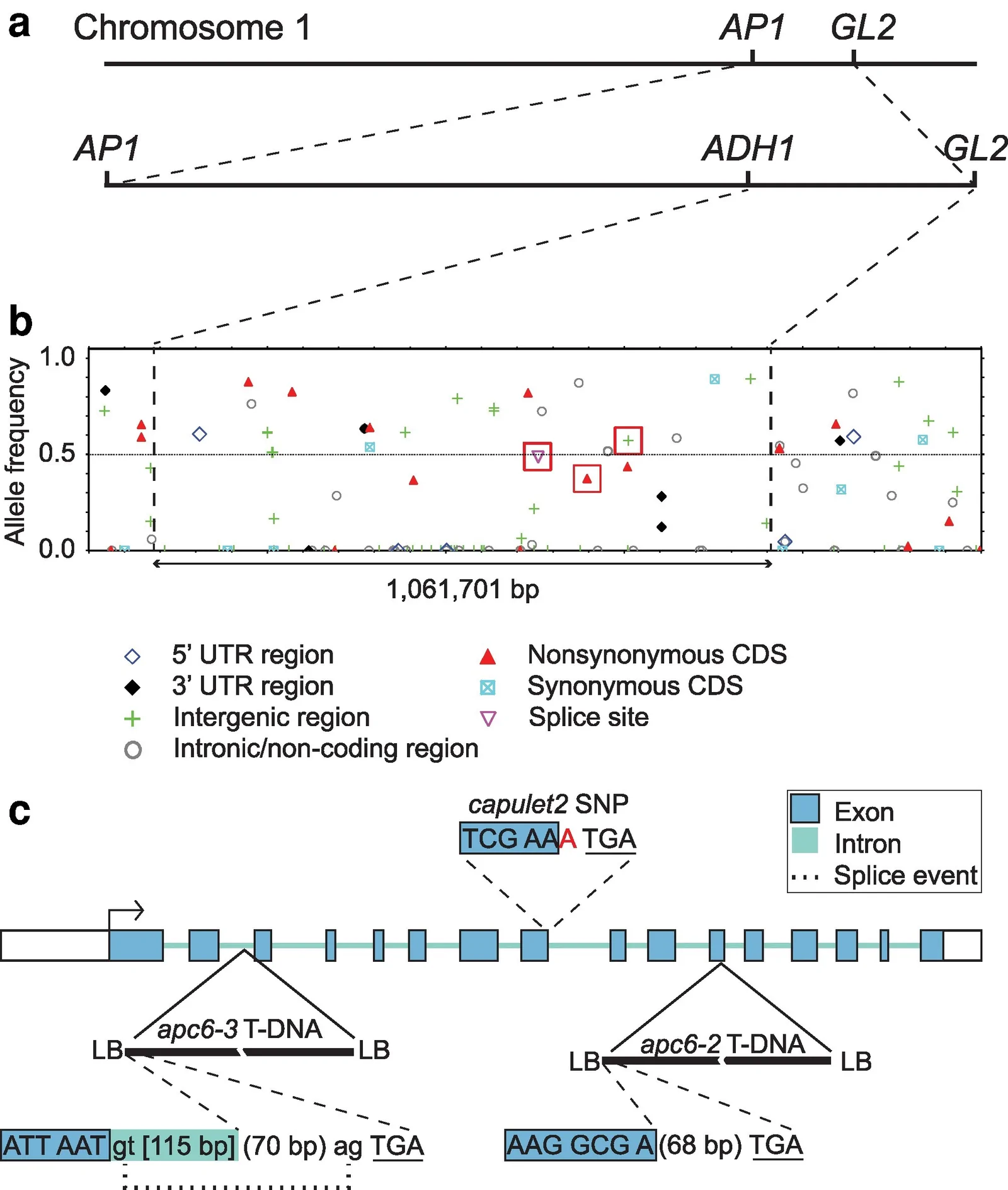
Identification of the causative *cap2* mutation. a) Identification of candidate causative SNPs located on chromosome 1 between the visible mutant markers *ap1* and *gl2* of *multiply marked chromosome 1* (*mm1*). Segregation analysis using molecular markers narrowed down the location of the *cap2* mutation between *ADH1* and *gl2*. b) SNP analysis of 12 independent comparisons between a *cap2/CAP2* and a *CAP2/CAP2* individual. Representation of identified SNPs, the type of mutation and allele frequency in a region of 1.06 Mbp of comparison 3. c) Molecular analysis of *cap2* and *apc* mutant alleles: The *cap2* G → A conversion (red) abolishes splicing and intron translation leads to a stop codon directly after the *cap2* SNP. The *apc6-2* T-DNA insertion, located in exon 12, has a 10 bp deletion and an in-frame stop codon 68 bp into the T-DNA. The *apc6-3* T-DNA insertion located in intron 2 has a 12 bp deletion. cDNA analysis revealed a cryptic splice acceptor site present in the T-DNA 70 bp inwards, resulting in splicing of intron 2 (up till the T-DNA insertion site) and part of the T-DNA, followed directly by an in-frame stop codon. Both *apc6-2* and *apc6-3* have at least 2 truncated T-DNA insertions at the same locus consecutively in opposite orientations. Exons and introns are highlighted by color and letter size (blue uppercase and green lowercase, respectively). Dotted line indicates splice event. Underlined nucleotides represent a stop codon. LB = T-DNA left border.

### *APC6* mutants are defective in endosperm development

We obtained 3 homozygous T-DNA insertion lines for *SRF5* and 2 T-DNA insertion lines flanking the intergenic candidate SNP and found that homozygous mutant plants for these insertions had normal seed sets and were indistinguishable from wild-type plants. Our genome sequencing analysis revealed that the *cap2* mutant carries a splice site mutation in intron 8 of *APC6*, and sequencing of *cap2* cDNA verified that this resulted in an unspliced transcript, introducing an early in-frame stop codon (Fig. 1c, Figure S2). We obtained 2 T-DNA insertion lines for *APC6*, 1 that we named *apc6-2* (SAIL_442_F11) carrying an insertion located in exon 12 that results in the formation of a premature stop codon and a 2nd named *apc6-3* (SALK_008789) that carries an insertion located in the 2nd intron, resulting in a cryptic splice acceptor site inside the T-DNA sequence leading to an in-frame stop codon (Fig. 1c). The predicted transcripts of both T-DNA lines were verified by sequencing of cDNA from the mutant alleles (Figure S2).

We found that when heterozygous *apc6-2* and *apc6-3* were crossed with wild-type (WT) pollen the resulting seeds displayed developmental defects that were apparent at 4 days after pollination (DAP) and that resembled the seed phenotype observed in *cap2/+* (Fig. 2a). To further characterize the endosperm phenotypes, we crossed *apc6-2/+* with an early endosperm (EE) green fluorescent protein (GFP) reporter line that shows GFP expression before endosperm cellularization and a total endosperm (TE1) GFP reporter line expressing GFP after endosperm cellularization (Bjerkan et al. 2023; van Ekelenburg et al. 2023). We found that a large proportion of seeds in *apc6-2/+*;*EE-GFP* plants had a reduced number of endosperm nuclei that were enlarged and marked by a strong EE-GFP signal at 4 DAP (Fig. 2a; Figure S3). In *apc6-2/+*;*TE1-GFP* plants, however, the postcellularization marker *TE1-GFP* was rarely detected in phenotypically mutant endosperm 4 DAP, suggesting that the mutant endosperm remain uncellularized (Figure S3).

**Figure 2:**
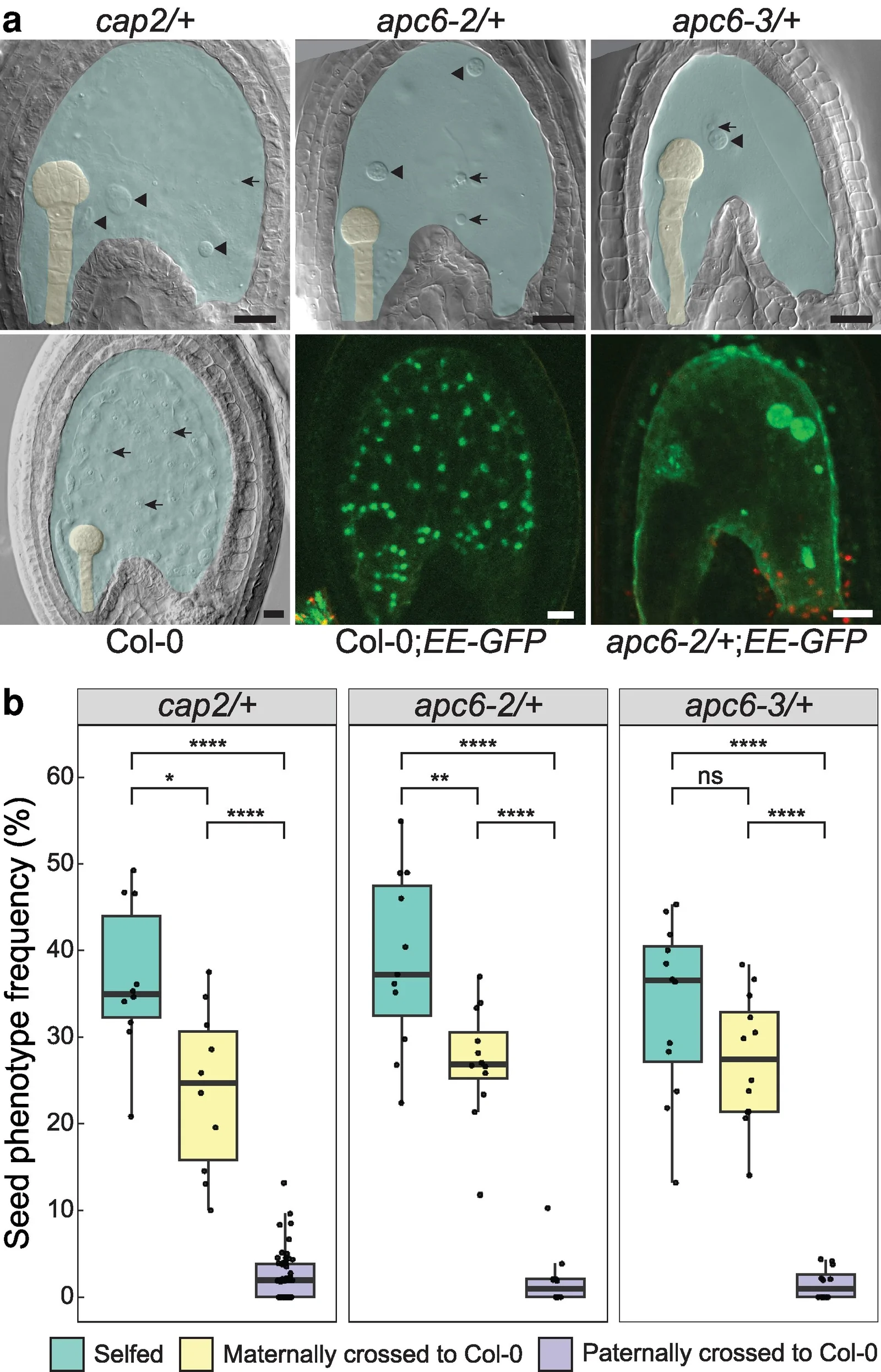
Seed phenotypes in *cap2*, *apc6-2* and *apc6-3* display gametophytic maternal effects. a) Endosperm phenotypes of *cap2/+*, *apc6-2/+* and *apc6-3/+* crossed maternally to Col-0 display arrested endosperm development at 4 days after pollination (DAP). A lower number of enlarged endosperm nuclei are observed compared to wild-type Col-0. Closed arrowheads indicate enlarged and abnormally shaped endosperm nuclei, arrows indicate smaller endosperm nuclei. A turquoise hue is added to highlight the endosperm, yellow hue highlights the embryo. Selfed *EE-GFP* compared to *apc6-2/+;EE-GFP* highlights enlarged and abnormally shaped endosperm nuclei. GFP-expressing seeds were counterstained with Propidium Iodide (red). Scale bar = 20 µm. b) Mutant seed phenotype frequencies for *cap2/+*, *apc6-2/+* and *apc6-3/+* at 4 DAP when selfed or crossed reciprocally to Col-0. n > 10 siliques per cross. Statistics was performed according to the Wilcoxon rank sum test; * *P* < 0.05, ** *P* < 0.01, **** *P* < 0.0001.

Notably, the Arabidopsis *nomega* mutant, caused by *Ds*-transposon insertion in *APC6* (Kwee and Sundaresan 2003) and an *APC6* mutant in rice (Awasthi et al. 2012) have both been reported to be female gametophytic mutants displaying prefertilization developmental defects. To investigate this discrepancy, we analyzed unfertilized ovules of wild-type, *cap2/+*, *apc6-2/+*, and *apc6-3/+*. We found that the embryo sac central cell and egg cell and the egg apparatus of mutant lines are indistinguishable from the WT (Table S2), and the 3 independent mutant alleles that we have identified for *APC6* show only postfertilization seed defects.

We previously reported that *cap2* is a female gametophyte mutant, because seed defects are only observed when the mutant allele is transmitted maternally (Grini et al. 2002). We carried out reciprocal crosses between *cap2/+*, *apc6-2/+*, and *apc6-3/+* and WT plants and quantified defects in seed development (Fig. 2b, Data S2). We found that seeds failed to develop only when mutant lines were used as female parents (24.2%, 27.3%, and 27.5% for *cap2/+*, *apc6-2/+*, and *apc6-3/+*, respectively) whereas seeds developed normally when wild-type plants were crossed with pollen from mutant lines. As initially reported for *cap2* (Grini et al. 2002), we did not find homozygous individuals in progenies of self-pollinated *apc6-2/+* and *apc6-3/+* plants (*n* = 47 and 42, respectively).

Collectively, these findings indicate that mutations on *APC6* result in a maternal gametophyte effect on seed development.

### Genetic complementation of mutants with *APC6*

To verify that *cap2* is caused by mutation of *APC6*, we transformed *cap2/+* and *apc6-3/+* plants with a *proAPC6:APC6-GFP* (APC6-GFP) transgene. The transgene was also transformed into WT L*er*-1 plants to confirm endosperm expression (Figure S4).

Plants from unique transformation events were genotyped for mutant alleles and APC6-GFP, and mutant seed phenotype frequencies were determined (Data S3). The frequency of mutant seeds was significantly reduced for the alleles tested, both in a hemizygous (*APC6-GFP/+*) or a homozygous (*APC6-GFP/APC6-GFP*) transgene background (Fig. 3; Figure S5). Notably, homozygous *cap2* genotypes were identified in the T2 generation (Fig. 3; Figure S6a). Several homozygous *apc6-3* individuals were detected in the T3 generation (Figure S6b) and no mutant seed phenotypes were observed (Figure S5). Homozygous *apc6-3* mutants in a homozygous rescue construct background crossed to *apc6-2*/+ plants demonstrated several homozygous *apc6-2* individuals in the F2, as well as trans-homozygous *apc6-2*/*apc6-3* (Figure S6c).

**Figure 3:**
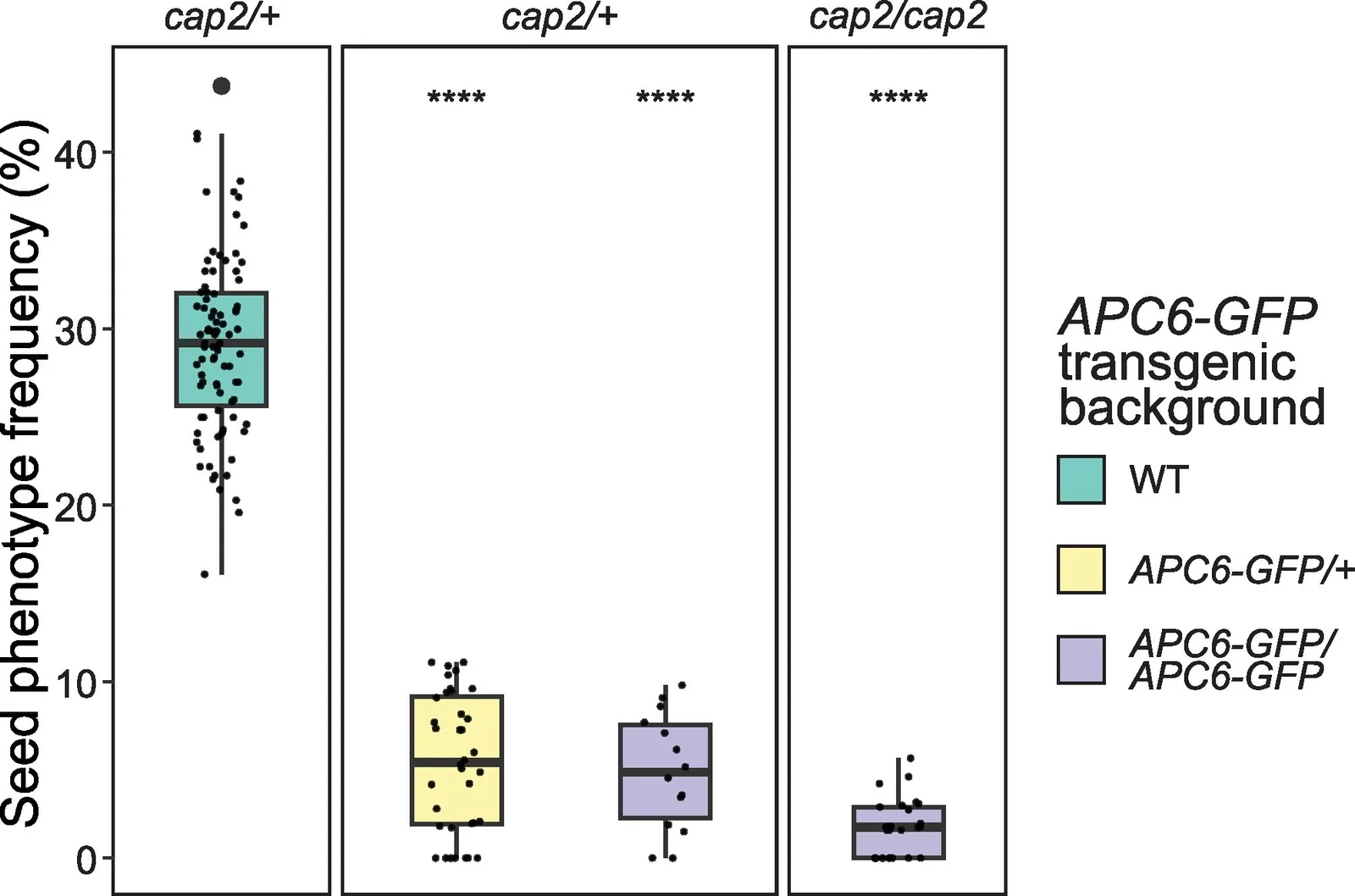
Complementation of *cap2* with an *APC6* transgene restores seed phenotype. Individuals heterozygous for the *cap2* SNP (*cap2*/*+*) were identified in both a hemizygous and homozygous transgenic background and homozygous individuals (*cap2*/*cap2*) were identified in a homozygous transgenic background. A significant reduction of the gametophyte maternal effect seed phenotype frequency is observed for *cap2*/+ in a hemizygous transgenic background. In a homozygous transgenic background, a significant reduction is observed for *cap2*/+ and *cap2/cap2*. Data from independent transgenic lines are merged for each box-plot: 4 independent rescue lines displayed significantly reduced phenotype frequency in a heterozygous *APC6-GFP* transgenic background, of which 3 segregated individuals with a homozygous construct background. Of these 3 lines, homozygous cap2/cap2 mutants were identified in 2. Statistics was performed according to the Wilcoxon rank sum test; **** *P* < 0.0001.

Collectively, these data show that all *cap2/apc6* mutants can be rescued by a APC6-GFP transgene.

### Maternal penetrance and transmission of mutant alleles

We found that when plants carrying the 3 different *APC6* mutant alleles were pollinated with WT pollen, >50% of the seeds showed developmental abnormalities (Fig. 2b), suggesting that these mutations were not fully penetrant. Therefore, we investigated in more detail the parental transmission of the different mutant alleles. To this aim, we selfed and crossed *cap2/+*, *apc6-2/+*, and *apc6-3/+* reciprocally with WT plants and determined the transmission of the mutant alleles by molecular genotyping (*cap2* and *apc6-3*) or T-DNA herbicide resistance (*apc6-2*) of seedlings (Fig. 4, Data S4). Paternal transmission of *cap2* (44.4%), *apc6-2* (47.4%), and *apc6-3* (46.7%) was not significantly different from the expected Mendelian transmission. Notably, the maternal transmission observed for *cap2* (30.5%), *apc6-2* (25.1%), and *apc6-3* (20.2%) was significantly lower than the expected 1:1 ratio when crossed to WT and also significantly lower than the expected 3:1 ratio in self crosses.

**Figure 4:**
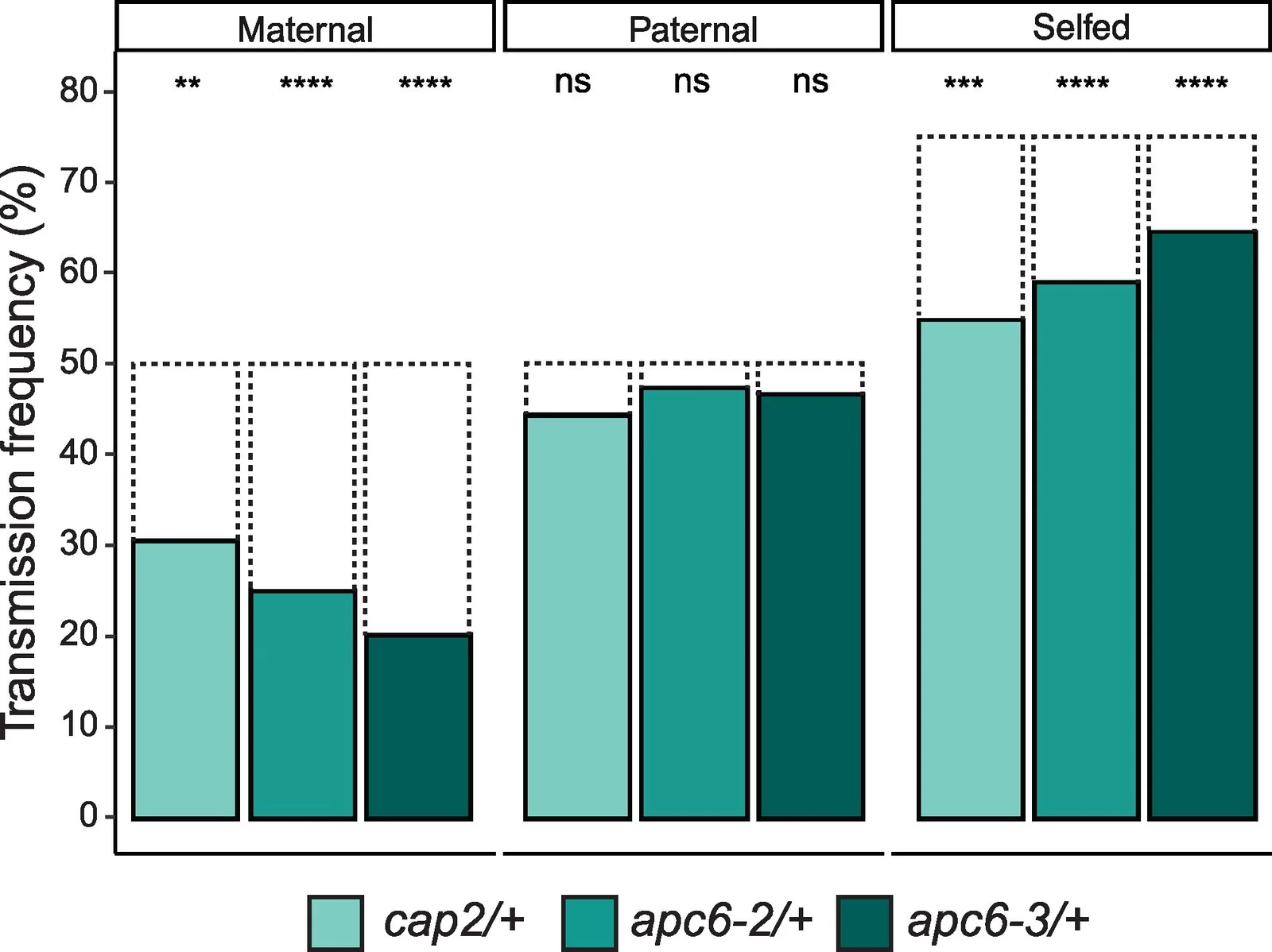
Genetic analysis shows reduced maternal transmission of *cap*2, *apc6-2* and *apc6-3*. Mutant alleles were selfed and crossed reciprocally to Col-0 and transmission was determined by genotyping (*cap2* and *apc6-3*) or using T-DNA herbicide resistance (*apc6-2*). Maternal transmission was significantly reduced while paternal transmission was not significantly different from the expected (50%, dashed line). Selfed transmission frequency for all mutant alleles was significantly lower than expected (75%, dashed line). Statistics was performed according to the Chi square test; ** *P* < 0.01, *** *P* < 0.001, **** *P* < 0.0001.

These data support the finding that maternal but not paternal *cap2/apc6* alleles have significantly reduced transmission frequencies, suggesting a maternal bias in the role of APC6.

### Expression of *APC6* increases with endosperm cellularization

We investigated *APC6* expression throughout seed development using publicly available RNA sequencing datasets (Bjerkan et al. 2020). As controls, we used several MADS-box transcription factors known to be expressed only in the endosperm, either bi-parentally or showing parental allelic bias (Colombo et al. 2008; Kang et al. 2008; Shirzadi et al. 2011). We found that *APC6* was highly expressed between 1 and 2 DAP, decreasing between 3 and 4 DAP and increasing again at 6 DAP (Figure S7).

Because *APC6* shows a dynamic pattern of expression during seed development, we carried out a phenotypic characterization at different stages of seed development (2, 4, 6, and 9 DAP) using *cap2/+*, *apc6-2/+*, and *apc6-3/+* plants crossed with wild-type Col-0 pollen. Generally, we found that both embryo and endosperm development was delayed compared to wild-type plants (Figure S8) and in this phenotypic class endosperm proliferation was arrested after a few rounds of nuclear division. We also observed another phenotypic class in which the endosperm was not fully cellularized at late stages in seed development (6 to 9 DAP), while embryo development was normal, although delayed, compared to development in wild-type plants (Figure S8). The occurrence of seed phenotypes and collapsed seeds at 4 DAP and 6 DAP was highly similar in all *APC6* mutants (Figure S8b, Data S5).

### *APC6* is a maternally expressed imprinted gene

Because all of the *APC6* mutants identified showed maternal gametophyte effects on endosperm development, we hypothesized that this finding could be caused by uniparental expression of *APC6* transcripts. To test this hypothesis, we performed reciprocal crosses between different *Arabidopsis* accessions (L*er*-1, Col-0, C24, and Tsu-1) and analyzed the parental allele-specific expression of *APC6* in developing seeds by taking advantage of SNPs between these accessions (Fig. 5a, Data S6). We found that at 4 DAP the expression of *APC6* was exclusively from the maternal allele in most of the accessions tested. We quantified the maternal:paternal allelic ratios for the different reciprocal crosses and found that the maternal allele was consistently expressed between 8- and 16-fold higher than the paternal allele (Fig. 5b, Data S6).

**Figure 5:**
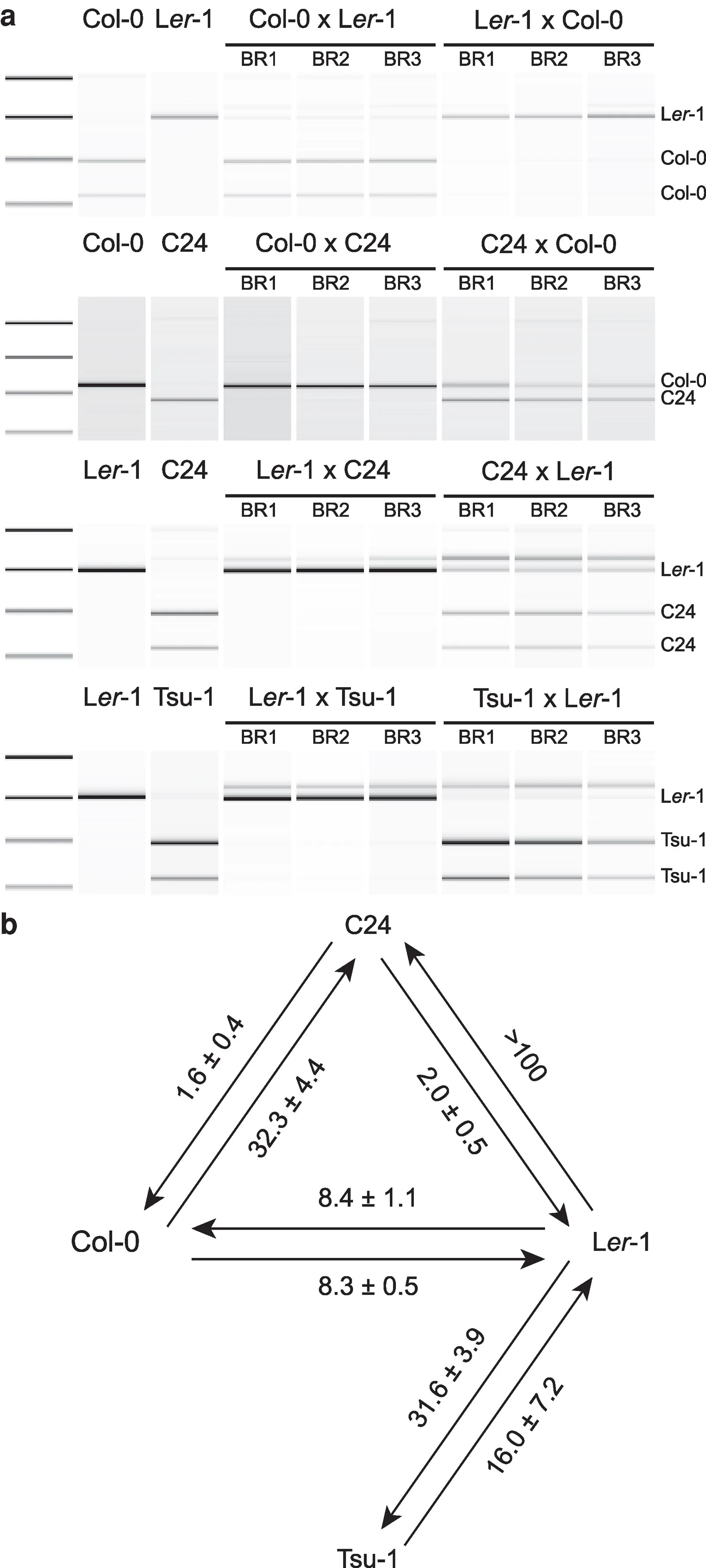
*APC6* is a maternally expressed imprinted gene. a) Imprinting analysis of *APC6* using accession-specific restriction digestion of SNPs in reciprocal crosses of Col-0, C24, L*er*-1 and Tsu-1. cDNA was generated from 4 days after pollination (DAP) crossed seeds. One replicate of homozygous wild-type controls is included as accession-specific cDNA reference and 3 biological replicates for each reciprocal cross are shown. One of 2 bioanalyzer replicates is presented and the accession of the maternal contributor is named first. In all directions, except for C24 as female parent, *APC6* is maternally expressed. b) Molarity ratios of maternal and paternal digested fragments from bioanalyzer profiles in a). Arrows point from maternal to paternal ecotype parent. Parental ratios are determined from all biological replicates and both bioanalyzer technical replicates. sd, standard deviation.

To verify these results, we analyzed RNA-seq data of early developing seeds (1 to 6 DAP) from reciprocal crosses between L*er*-1 and Col-0 (Shirzadi et al. 2011; Bjerkan et al. 2020). We identified several parental allele-specific SNPs, which we used to determine parental allele expression frequencies (Fig. 6, Data S7). As controls we used MADS-box transcription factors, known to be expressed exclusively in the endosperm and biparentally (*AGL62*), maternally biased (*AGL36*) or paternally biased (*AGL23*) (Colombo et al. 2008; Kang et al. 2008; Shirzadi et al. 2011). We found that during early stages of seed development (1 to 4 DAP), *APC6* displays a strong maternal expression bias, similar to *AGL36*. The maternal bias of *APC6* was highly concordant with the molarity ratios determined experimentally (11.1-fold and 8.4-fold, respectively; Data S7). However, whereas *AGL36* expression remains maternally biased at 6 DAP, the paternal expression of *APC6* increased, which could be attributed to an increasing contribution of the developing embryo.

**Figure 6:**
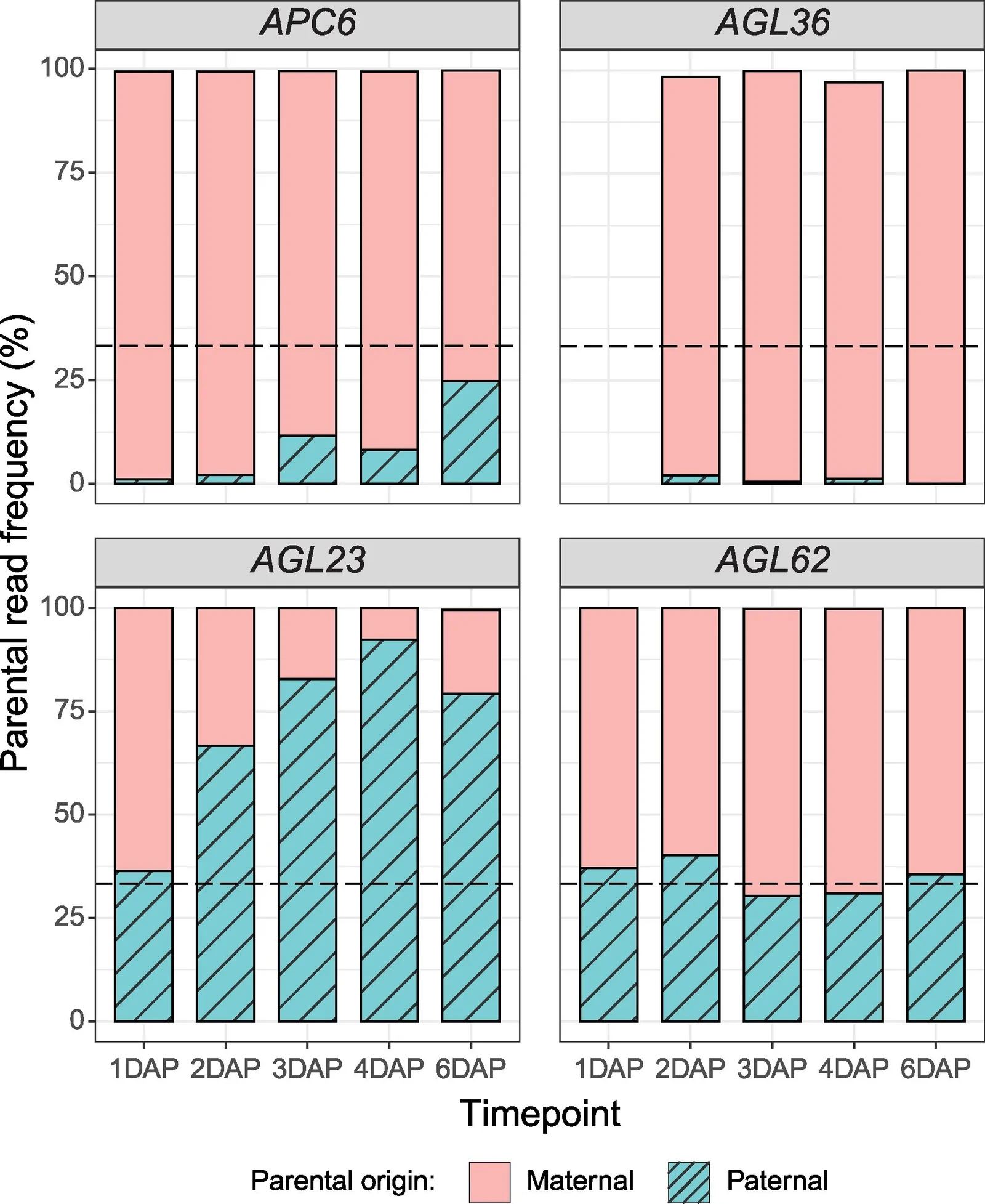
Seed stage analysis of parent specific allelic expression of *APC6* and imprinting control genes. Frequency of reads mapped to parental alleles of *APC6*, *AGL36* (MEG control), *AGL23* (PEG control) and *AGL62* (BEG control) at different timepoints (one, 2, 3, 4 and six days after pollination (DAP)). Maternal:paternal read frequency ratios are plotted at respective timepoints. SNP analysis was performed with Geneious Prime version 2021.1.1 using 0.2 as variant (L*er*-1) frequency threshold (except *AGL23*—0.05 variant frequency threshold) and SNPs were weighted according to their coverage.

We hypothesized that the silencing of the paternal *APC6* allele may be caused by DNA methylation—a common characteristic of *Arabidopsis* MEGs (Gehring and Satyaki 2017; Hornslien et al. 2019; Batista and Köhler 2020). To test this hypothesis, we pollinated WT L*er*-1 plants with pollen from *met1-7/+* and *drm1-2*;*drm2-2*;*cmt3-11* (*ddc/+*) plants (Figure S9a and b respectively). Due to possible DNA methyltransferase redundancy (Zhang and Jacobsen 2006), we also generated a quadruple *met1-7*;*drm1-2*;*drm2-2*;*cmt3-11* (*ddcm/+*) mutant and used as pollen donor (Figure S9c). Depletion of DNA methylation in the pollen, using single, triple or quadruple mutants did not reactivate the silenced paternal expression of *APC6* (Figure S9a–c). Notably, the maternal:paternal expression ratios for all methyltransferase crosses were similar to the WT controls (Fig. 5b, Figure S9d).

Collectively, these data suggest that *APC6* is a maternally expressed imprinted gene and that imprinting of *APC6* is not mediated by canonical DNA methylation.

### Maternal expression of the APC/C

The APC/C is a large, multisubunit complex consisting of at least 14 subunits in *Arabidopsis*. These subunits can be divided into groups based on their structure and function, such as scaffolding, catalytic, substrate recognition and platform units (Eloy et al. 2015). APC6 is a scaffolding unit consisting of tetratricopeptide repeats (TPRs), such as APC3a and b, APC7, and APC8. In order to test whether members of other functional groups of the APC/C also display a maternal preference, we investigated *APC1*, a platform subunit containing proteasome-cyclosome (PC) repeats. APC1 is the largest subunit of the APC/C and has previously been described to be important for female gametogenesis and embryogenesis (Wang et al. 2013).

Heterozygous *apc1-2/+* mutants displayed abnormalities in embryo sac formation, but no apparent reduction in seed set was found (Figure S10a, Data S8). When *apc1-2/+* was crossed maternally to Col-0, gametophyte maternal effect endosperm phenotypes highly reminiscent of cap2*/apc6* were found, both in single *apc1-2/+* mutants and in an *apc1-2/+;apc6-2/+* double mutant (Fig. 7a). Corresponding to mutants of *apc6*, *apc1-2/+* mutants displayed endosperm defects only when crossed maternally to Col-0, with a phenotype frequency of 37.1% (Fig. 7b).

**Figure 7:**
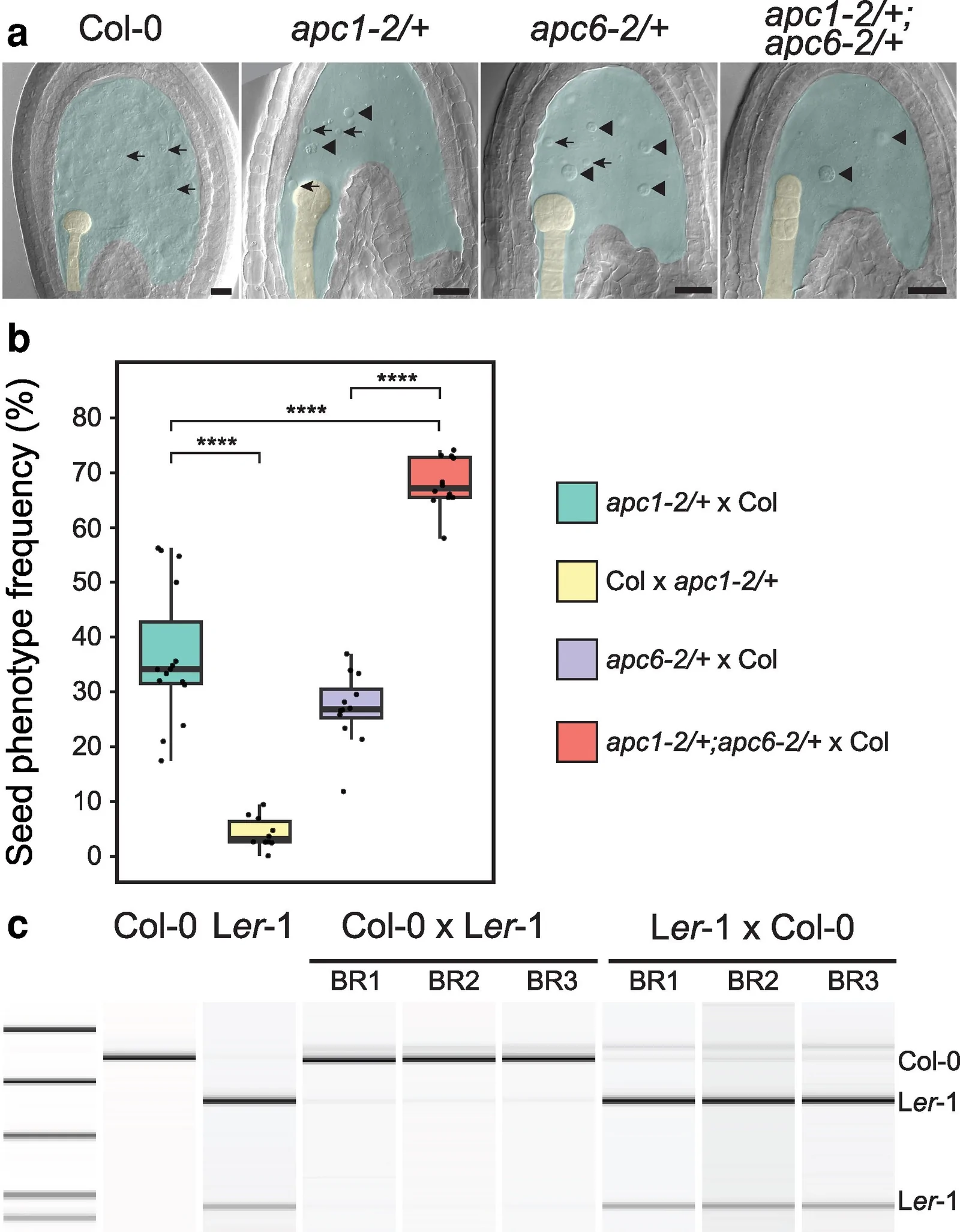
*APC1* mutants display a *cap2/apc6* endosperm phenotype and is a maternally expressed imprinted gene. a) Seed phenotypes of *apc1-2/+* and *apc1-2/+;apc6-2/+* double mutant at 4 days after pollination (DAP) when crossed maternally to Col-0. A turquoise hue is added to highlight the endosperm, yellow hue highlights the embryo. Arrows indicate small nuclei, arrowheads indicate large nuclei. Scale bar = 20 µm. b) Seed phenotype frequencies of *apc1-2/+* crossed reciprocally to Col-0 and *apc6-2/+* and *apc1-2/+;apc6-2+* crossed maternally to Col-0 at 4 DAP. No seed phenotype is present when *apc1-2/+* is crossed paternally, significantly different from when it is crossed maternally. The phenotype frequency of maternally crossed *apc1-2/+;apc6-2/+* double mutants is significantly increased compared to both single mutants. Statistics was performed according to Wilcoxon rank sum test; **** *P* < 0.0001. c) Imprinting analysis of *APC1* using accession-specific restriction digestion of SNPs in reciprocal crosses between Col-0 and L*er*-1 at 4 DAP. One replicate of each homozygous wild-type control and 3 biological replicates for the reciprocal crosses are shown. One of 2 bioanalyzer replicates is presented and the accession of the maternal cross partner is named first. Mostly maternal *APC1* expression was found in both directions of the reciprocal crosses shown.

When crossing the *apc1-2/+;apc6-2/+* double mutant maternally to Col-0 we observed a synergistic epistatic effect in which the phenotype frequency of the double mutant (67.1%) was significantly increased compared to both single mutants (Fig. 7b). In addition, transmission of mutant alleles from a selfing *apc1-2/+;apc6-2/+* double mutant was investigated. Using the phenotype frequencies of *apc1-2/+* and the transmission frequency of *apc6-2/+*, we calculated the expected transmission frequencies to be 60.9% for the *apc6-2* allele and 34.9% for the *apc1-2* and *apc6-2* alleles together (Data S8). Notably, transmission of the *apc6-2* allele deviated from this expectancy, with a significant decrease in offspring carrying the mutant allele (57.4%; Figure S10b). However, the transmission of both mutant alleles together was not significantly different from our expectation.

Last, we investigated whether *APC1* expression was maternally biased by genomic imprinting. To this end, we analyzed the parental allele-specific expression of *APC1* in developing seeds using different *Arabidopsis* accessions (L*er*-1, Col-0, C24, and Tsu-1) as described previously. In 4 DAP seeds, *APC1* was exclusively expressed from the maternal allele in all accessions and reciprocal crosses (Fig. 7c, Figure S10c).

Collectively, these data suggest that both *APC1* and *APC6* are maternally expressed imprinted genes that are required maternally both in the scaffolding and platform units of the APC/C for early endosperm development, suggesting a maternal bias in the role of the APC/C complex.

## Discussion

In this study, we identified that mutation in *APC6* is associated with the female gametophyte maternal effect mutant *capulet2* (*cap2*). Molecular characterization of *cap2* has revealed that *APC6* is a maternally expressed imprinted gene implicated in endosperm proliferation and cellularization. Our data reveals that the maternal contribution of the APC/C is critical for endosperm development in plants.

### *Capulet2* causative mutation is located in *APC6*

We have found that *cap2* is caused by a splice donor mutation in *APC6*. We were able to functionally complement *cap2, apc6-2* and *apc6-3* mutants with an *APC6* transgene, allowing the generation of homozygous *cap2, apc6-3*, and *apc6-2/apc6-3* mutant plants. Previous studies have revealed that mutations on several APC/C subunits result in female gametophyte defects (Saleme et al. 2021), including Arabidopsis *nomega*, that carry a *Ds-*transposon insertion in *APC6* (Kwee and Sundaresan 2003) and a mutant in rice *OsAPC6* (Awasthi et al. 2012). We were unable to retrieve the original *nomega* mutant line and can therefore only speculate on the basis of the reported discrepancy. Under our growth conditions, 3 APC6 mutant alleles—*cap2*, *apc6-2*, and *apc6-3*—exhibit defects exclusively during early seed development. Female gametophytes carrying *cap2*, *apc6-2*, and *apc6-3* were indistinguishable from the wild type. Although the *nomega* mutation introduces an earlier translational stop than the alleles characterized here, this mutation is unlikely to account for the phenotypic differences as we did not detect any variation in female gametophyte phenotypes throughout our allelic series. Because our experiments employed different accessions and growth temperatures than those used in the *nomega* study, we speculate that the discrepancy may arise from accession-specific genetic background effects or temperature dependent properties of the alleles. Both factors present promising avenues for future investigation into APC function.

### *APC6* is a maternally expressed imprinted gene

Our analysis, using both allele-specific assays and RNA-seq allele-specific expression quantification has revealed that *APC6* is an imprinted, maternally expressed gene. However, since we used whole seeds in the analysis, transcripts from the maternal seed coat or embryo could affect this interpretation (Schon and Nodine 2017). To address this caveat, we analyzed publicly available expression data from early developing seeds, which showed that *APC6* is 6-fold more highly expressed in the endosperm than the seed coat (Belmonte et al. 2013) and 2.7-fold more highly expressed in the endosperm than in all other seed tissues (Martin et al. 2026). Due to the different genotypes of the endosperm, embryo, and seed coat (2:1; 1:1; and 2:0 maternal:paternal, respectively), a whole seed maternal:paternal ratio of approximately 2:1 can be estimated if *APC6* is equally expressed from both parental alleles. The maternal:paternal ratios in our analyses are at least >4-fold higher, ranging up to >50-fold higher, indicating that expression of *APC6* is maternally biased in the endosperm. Our data also indicate that imprinting of *APC6* is accession specific, a phenomenon that has been observed in previous studies of imprinted genes (Wolff et al. 2011; Pignatta et al. 2014).

It has been proposed that MEGs are regulated by DNA methylation through the activity of MET1 that silences gene expression in gametes and by DEMETER (DME) that removes methylation in the central cell and endosperm, thus restoring the expression of maternal alleles (Choi et al. 2002; Xiao et al. 2003; Gehring et al. 2006). Here, we have investigated the role of DNA methylation in imprinting of *APC6* by crossing L*er*-1 with various DNA methyltransferase mutants in a Col-0 background and performed SNP digestion analysis to determine if expression of the paternal allele is reactivated. The single, triple and quadruple mutants of *met1-7*, *drm1-2*;*drm2-2*;*cmt3-11* and *met1-7;drm1-2*;*drm2-2*;*cmt3-11* were used to reduce redundancy by other methyltransferases. None of the mutant combinations showed an increased paternal expression and thus we concluded that imprinting of *APC6* is not caused by these canonical DNA methyltransferases. This finding is consistent with previous reports in which the majority of MEGs was not regulated by MET1 DNA methylation maintenance (Hornslien et al. 2019). Further analysis of the epigenomic landscape of *APC6* is thus required to determine if *APC6* imprinting is guided by histone modifications or RNA dependent DNA methylation.

It is experimentally demanding to distinguish de novo transcripts after fertilization from transcripts originating from the central cell as maternal carry over (Evans and Kermicle 2001). The *APC6* expression profile is constructed from whole seed RNA (Figure S7), and RNA originating from the seed coat or the embryo is present as discussed above. Compared to the endosperm-specifically expressed *AGL36*, *AGL23*, and *AGL62*, overall expression of *APC6* is higher at the early stages (1 and 2 DAP) of seed development and carry over of transcripts from the maternal central cell, as described on a general basis (Luo et al. 2014) cannot be excluded. However, at 4 DAP, allele-specific expression is mostly maternal, supporting the finding that *APC6* is imprinted. Long-lived mRNAs in the endosperm have been described for endosperm maturation phase transcripts (Matilla 2022), but it seems unlikely that transcripts, originating from the maternal central cell, are abundant at the globular embryo stage. Moreover, microarray data (Belmonte et al. 2013) and recent single nuclei sequencing of seed tissue (Martin et al. 2026) indicated that expression of *APC6* at the globular embryo stage is higher in the endosperm compared to the total of seed coat and embryo expression. Additionally, the increase in paternal expression at 6 DAP could be due to a stronger impact of the growing embryo, as this coincides with consumption of the endosperm (Nowack et al. 2010; Lafon-Placette and Köhler 2014). As such, late-stage expression of *APC6* is directed toward a parental expression ratio closer to the 1:1 diploid genotype of the embryo. Altogether, the findings of this study indicate that *APC6* is a maternally expressed imprinted gene in the endosperm.

### The APC/C is maternally biased

The APC/C is a large protein complex consisting of at least 14 core subunits and is essential for gamete development (Saleme et al. 2021). Genetic analyses have revealed that several subunits of the APC/C are essential for megagametogenesis (*APC2*, *APC3a/APC3b*, and *APC10* [Capron et al. 2003; Pérez-Pérez et al. 2008; Eloy et al. 2011]) and male gametogenesis (*APC8* and *APC13* [Saze and Kakutani 2007; Zheng et al. 2011]). Furthermore, overexpression of *APC8* displayed reduced seed sets, suggesting that careful dosage regulation of *APC* subunits is essential for seed development (Zheng et al. 2011). Here, we have demonstrated that both *APC6* and *APC1* are imprinted and expressed predominantly from the maternal genome. When mutated, both APC/C loci lead to a gametophyte maternal effect phenotype in the developing endosperm, and double mutants suggest an additive effect on seed survival. The strong maternal expression bias of *APC1* in all tested ecotypes further emphasizes the importance of maternal control of the APC/C during early seed development. Interestingly, and in addition to *APC6* and *APC1* mutants, *APC4* and *APC11* also show gametophyte maternal effect phenotypes (Kwee and Sundaresan 2003; Wang et al. 2012, 2013; Guo et al. 2016), in line with the notion of APC/C being under maternal control.

It remains unclear why subunits of such a crucial protein complex are subjected to parental allele-specific regulation, and the function of genomic imprinting in this regard remains to be determined. Our data support the gene dosage hypothesis for the evolution of genomic imprinting, which states that imprinting evolved as a mechanism to control and regulate gene expression levels (Dilkes and Comai 2004; Ferguson-Smith 2011). For subunits of the APC/C, in which too little or too much expression of a gene is detrimental for seed development, genomic imprinting could be a measure to balance gene expression contributing to this protein complex. Taken together, our observations propose a maternal bias to the control of endosperm proliferation.

## Materials and methods

### Plant material and growth conditions

Wild-type (WT) accessions Columbia (Col-0), Landsberg *erecta* (L*er*-1), Tsushima (Tsu-1), C24 and T-DNA mutant lines were obtained from the Nottingham Arabidopsis Stock Centre (NASC; Scholl et al. 2000). Seedlings were grown on MS-2 (Murashige and Skoog medium with 2% sucrose) plates in a 16-h-light/8-h-dark cycle at 22 °C for 10 days prior to transferring to soil. Plants were further grown in a 16-h-light/8-h-dark cycle at 18 °C. The T-DNA mutant plant lines *apc6-2* (SAIL_442_F11), *apc6-3* (SALK_008789), *apc1-2* (SALK_059826; g), SALK_002024C (*SRF5*), SALK_070429C (*SRF5*), SAIL_1280_D04 (*SRF5*), SALK_120562 (intergenic), SALK_033225 (intergenic), *cmt3-11* (SALK_148381 [Chan et al. 2006]), *met1-7* (SALK_076522 [Kanno et al. 2008]), and *drm1-2;drm2-2* (N16383 [Chan et al. 2006]) were in the Col-0 accession background (except *apc6-3*; Col-3). The triple *drm1-2*;*drm2-2*;*cmt3-11* mutant was crossed with the hemizygous *met1-7* mutant to generate a hemizygous *met1-7*;*drm1-2*;*drm2-2*;*cmt3-11* quadruple mutant. Methyltransferase mutants were maintained as hemizygous (Mathieu et al. 2007). Endosperm marker lines used in crosses with *apc6-2*, proAT5G09370>>H2A-GFP (EE-GFP) and proAT4G00220>>H2A-GFP (TE1-GFP) were in the Col-0 accession background (van Ekelenburg et al. 2023). For all crosses, closed flower buds were emasculated 2 days prior to crossing to avoid self-pollination.

### Microscopy

Crossed siliques were manually dissected and seeds were mounted on a microscopy slide in a clearing solution of glycerol and chloral hydrate as previously described (Grini et al. 2002). Microscopic analyses were performed using an Axioplan2 Imaging microscope equipped with a Zeiss Axio cam HDR camera. For fluorescent phenotypic analyses, seeds were mounted on a microscopy slide in 2 drops of tap water or 0.1% Tween in 1 × phosphate buffered saline (PBS; pH 7.4) or 30% glycerol with 20 μg/mL propidium iodide (PI). Seeds were analyzed for GFP fluorescence using an Andor DragonFly Spinning Disk confocal microscope with a 488 nm wavelength diode laser (Oxford) and imaged with a Zyla4.2 sCMOS camera (Oxford).

### DNA isolation and whole genome sequencing

Leaf and flower tissue from 12 individual *cap2/CAP2* (*cap2*mut) and 12 *CAP2/CAP2* (*cap2*wt) plants was collected in duplicate in a 2-mL round bottom tube containing a 5 mm metal bead and flash-frozen in liquid nitrogen. The frozen tissue was ground using a Retsch homogenizer for 1 min at 30 s^−1^. Genomic DNA (gDNA) was isolated as described in the ChargeSwitch gDNA Plant Kit (Invitrogen) manual and gDNA was eluted in 40 μL 10 mM Tris buffer (pH 8.5 at room temperature). Isolated gDNA was quality checked by Nanodrop and DNA concentrations were determined with the Qubit dsDNA BR Assay Kit (Life Technologies). DNA libraries were prepared using the KAPA Hyper Kit (Roche) with 24 Unique Dual Indexes (Illumina) and sequenced 150 bp paired-end over 2 lanes on the Illumina HiSeq 4000 system.

### Identification of the *cap2* causative single nucleotide polymorphism

Sequencing reads were preprocessed using fastp with default settings and the overrepresentation analysis parameter to remove reads with low quality score, irregular GC content, short length, and present sequencing adapters (Chen et al. 2018). Trimmed reads were then quality checked with outputs from fastp to ensure that trimming had been successful. Trimmed reads were mapped to the TAIR10 reference genome using bowtie2 (Langmead and Salzberg 2012). Variant calling was performed on aligned and sorted BAM files using samtools mpileup and piped to bcftools to produce VCF files (Li et al. 2009). VCF files were converted to SHOREmap format and analyzed using SHOREmap backcross and then annotated to output tables and plots of backcross SNPs (Sun and Schneeberger 2015).

### RNA isolation and cDNA synthesis

RNA was isolated from plant tissue (seeds, seedlings or flowers) as described in the Spectrum Total Plant RNA Kit and On-Column DNaseI Digestion Set (Sigma Aldrich) manual. The tubes were shaken in a MagNA Lyser Instrument (Roche) at 7000 rpm for 15 sec, centrifuged at 13,000 rpm in 4 °C for 15 sec, and then placed at −20 °C for 2 min. This procedure was repeated 3 times before proceeding with the protocol. cDNA was synthesized from 2.5 µL RNA as described in the SuperScript III Reverse Transcriptase (Invitrogen) manual and cDNA was purified using Wizard SV Gel and PCR Clean-Up System (Promega). Verification of transcripts from mutant alleles was performed by sequencing of PCR products amplified from cDNA as specified in Table S3.

### Single nucleotide polymorphism analysis

Single nucleotide polymorphisms (SNPs) between accessions were identified using GabiPD GreenCards (The GABI Primary Database) or Polymorph 1001 (1001 Genomes) and SNPs were verified by sequencing. Parental-specific expression based on these SNPs was determined by restriction digestion. For *APC1*, an SNP at position 6551 (G in L*er*-1; A in Col-0, Tsu-1 and C24) gains a restriction site for *AlwI* in the L*er*-1 accession. For *APC6*, an SNP at position 1297 (C in L*er*-1; G in Col-0, Tsu and C24) results in the loss of the restriction recognition site for *SacII* for the L*er*-1 accession. No restriction recognition site was identified covering the SNP at position 1516 (T in Col-0; A in C24), and therefore a recognition site for *BgIII* was introduced in the C24 accession using the Derived Cleaved Amplified Polymorphic Sequences (dCAPS) Finder 2.0 tool (Neff et al. 2002). The WT accessions Col-0, L*er*-1, Tsu-1, and C24 were crossed reciprocally and L*er*-1 was crossed maternally to *met1-7/+*, *ddc/+*, and *ddcm/+*. Siliques were dissected at 4 DAP using a stereomicroscope, and seeds harvested in MagNA Lyser Green Beads tubes (Roche) tubes were collected into liquid nitrogen. Seeds from 3 mother plants, 4 siliques each, were pooled for each biological replicate. RNA isolation and cDNA synthesis were performed as described above.

The SNP-containing DNA region was amplified by PCR with TaKaRa Ex Taq DNA Polymerase on 50 ng cDNA template using specific primers (Table S3), and PCR products were verified by sequencing. Restriction digestion was performed on a 400 ng PCR product with 10 U of enzyme in a final volume of 25 µL. Equal amounts of digested fragments were analyzed using DNA-1000-LabOnChip and 2100 Bioanalyzer (Agilent Technologies).

### Molecular cloning and genotyping

Genotyping was performed using the KAPA3G Plant PCR kit (Roche) and gene- and T-DNA–specific primers (Table S3). PCR products were sequenced by Eurofins Genomic and analyzed using Geneious software. To construct the *proAPC6:APC6-GFP* plasmid, a genomic fragment of the *APC6* gene, including 2 kb of the 5′-upstream sequence, was amplified by PCR and cloned between the AscI and SmaI sites of the pGFP_NOSG vector (Iwase et al. 2017). The resulting construct was then recombined into pGWB601 vector (Nakamura et al. 2010) using LR Clonase II. Genetic constructs were transformed into *Agrobacterium tumefaciens* C58 strain and introduced into *capulet2*, *apc6-3* and WT accession L*er*-1 using the floral dip method (Clough and Bent 1998).

### RNA sequencing analysis

Raw reads from RNA Seq data were obtained from online resources (Bjerkan et al. 2020). Trimmed reads were obtained using Cutadapt version 1.18 (Martin 2011), version TrimGalore 0.6.2 (Krueger 2012), and mapped to the Araport11 CDS reference transcriptome (Cheng et al. 2017) using Bowtie version 2.3.5.1 (Langmead and Salzberg 2012) with the parameters *–no-unal –no-mixed –no-discordant –sensitive –end-to-end -k 1*. Libraries were normalized using DESeq2 version 1.30.1 (Love et al. 2014). Statistical analysis was performed using “plyr” version 1.8.6 (Wickham 2011) and visualized using RStudio with “ggplot2” version 3.3.5 (Wickham 2016).

For the analysis of parental ratios, mapped reads were extracted using SAMtools version 1.9 (Li et al. 2009), and SNP counting was performed in Geneious Prime version 2021.1.1 (http://www.geneious.com/) using 0.2 as the variant (L*er*-1) frequency threshold (except *AGL23*—0.05 variant frequency threshold). Reads from all timepoints were merged and aligned to the Col-0 reference sequence. SNP counting was performed to present an overview of reliable SNPs between L*er*-1 and Col-0 for each gene. The allele frequencies of each SNP for each timepoint (>10 coverage per SNP) were weighted for the coverage and visualized in Rstudio with “ggplot2” version 3.3.5 (Wickham 2016) and “ggpattern” version 0.2.0 (Mike and Davis 2022).

### Image analysis and figure preparation

Images were processed using Fiji (Schindelin et al. 2012). Figures were assembled in Adobe Illustrator 2022 (Adobe Systems Incorporated, San Jose, USA).

### Accession numbers

All sequences generated in this study have been deposited to the National Center for Biotechnology Information Sequence Read Archive (https://www.ncbi.nlm.nih.gov/sra) with project number PRJNA808889.

## Supplementary Material

kiag647_Supplementary_Data

## Data Availability

All sequences generated in this study have been deposited to the National Center for Biotechnology Information Sequence Read Archive (https://www.ncbi.nlm.nih.gov/sra) with project number PRJNA808889.

## References

[kiag647-B1] Awasthi A et al 2012. Abnormal endosperm development causes female sterility in rice insertional mutant OsAPC6. Plant Sci. 183:167–174. 10.1016/j.plantsci.2011.08.007. 22195590

[kiag647-B2] Barton SC, Surani MA, Norris ML. 1984. Role of paternal and maternal genomes in mouse development. Nature. 311:374–376. 10.1038/311374a0. 6482961

[kiag647-B3] Batista RA, Köhler C. 2020. Genomic imprinting in plants—revisiting existing models. Genes Dev. 34:24–36. 10.1101/gad.332924.119. PMC6938664 31896690

[kiag647-B4] Belmonte MF et al 2013. Comprehensive developmental profiles of gene activity in regions and subregions of the Arabidopsis seed. Proc Natl Acad Sci U S A. 110:E435–E444. 10.1073/pnas.1222061110. PMC3562769 23319655

[kiag647-B5] Birchler JA . 1993. Dosage analysis of maize endosperm development. Annu Rev Genet. 27:181–204. 10.1146/annurev.ge.27.120193.001145. 8122901

[kiag647-B6] Bjerkan KN et al 2020. Genetic variation and temperature affects hybrid barriers during interspecific hybridization. Plant J. 101:122–140. 10.1111/tpj.14523. 31487093

[kiag647-B7] Bjerkan KN, Alling RM, Myking IV, Brysting AK, Grini PE. 2023. Genetic and environmental manipulation of Arabidopsis hybridization barriers uncovers antagonistic functions in endosperm cellularization. Front Plant Sci. 14:1229060. 10.3389/fpls.2023.1229060. PMC10433385 37600172

[kiag647-B8] Cao X, Jacobsen SE. 2002. Role of the arabidopsis DRM methyltransferases in de novo DNA methylation and gene silencing. Curr Biol. 12:1138–1144. 10.1016/S0960-9822(02)00925-9. 12121623

[kiag647-B9] Capron A et al 2003. The Arabidopsis anaphase-promoting complex or cyclosome: molecular and genetic characterization of the APC2 subunit. Plant Cell. 15:2370–2382. 10.1105/tpc.013847. PMC197302 14508008

[kiag647-B10] Chan SW-L et al 2006. RNAi, DRD1, and histone methylation actively target developmentally important non-CG DNA methylation in arabidopsis. PLoS Genet. 2:e83. 10.1371/journal.pgen.0020083. PMC1472700 16741558

[kiag647-B11] Chen S, Zhou Y, Chen Y, Gu J. 2018. Fastp: an ultra-fast all-in-one FASTQ preprocessor. Bioinformatics. 34:i884–i890. 10.1093/bioinformatics/bty560. PMC6129281 30423086

[kiag647-B12] Cheng C-Y et al 2017. Araport11: a complete reannotation of the Arabidopsis thaliana reference genome. Plant J. 89:789–804. 10.1111/tpj.13415. 27862469

[kiag647-B13] Chettoor AM, Phillips AR, Coker CT, Dilkes B, Evans MMS. 2016. Maternal gametophyte effects on seed development in maize. Genetics. 204:233–248. 10.1534/genetics.116.191833. PMC5012389 27466227

[kiag647-B14] Choi Y et al 2002. DEMETER, a DNA glycosylase domain protein, is required for endosperm gene imprinting and seed viability in arabidopsis. Cell. 110:33–42. 10.1016/S0092-8674(02)00807-3. 12150995

[kiag647-B15] Clough SJ, Bent AF. 1998. Floral dip: a simplified method forAgrobacterium-mediated transformation ofArabidopsis thaliana. Plant J. 16:735–743. 10.1046/j.1365-313x.1998.00343.x. 10069079

[kiag647-B16] Colombo L et al 1997. Downregulation of ovule-specific MADS box genes from Petunia results in maternally controlled defects in seed development. Plant Cell. 9:703. 10.2307/3870426. PMC156950 9165748

[kiag647-B17] Colombo M et al 2008. AGL23, a type I MADS-box gene that controls female gametophyte and embryo development in Arabidopsis. Plant J. 54:1037–1048. 10.1111/j.1365-313X.2008.03485.x. 18346189

[kiag647-B18] Dilkes BP, Comai L. 2004. A differential dosage hypothesis for parental effects in seed development. Plant Cell. 16:3174–3180. 10.1105/tpc.104.161230. PMC535866 15579806

[kiag647-B19] Eloy NB et al 2011. The APC/C subunit 10 plays an essential role in cell proliferation during leaf development. Plant J. 68:351–363. 10.1111/j.1365-313X.2011.04691.x. 21711400

[kiag647-B20] Eloy NB, de Freitas Lima M, Ferreira PCG, Inzé D. 2015. The role of the anaphase-promoting complex/cyclosome in plant growth. CRC Crit Rev Plant Sci. 34:487–505. 10.1080/07352689.2015.1078613.

[kiag647-B21] Evans MM, Kermicle JL. 2001. Interaction between maternal effect and zygotic effect mutations during maize seed development. Genetics. 159:303–315. 10.1093/genetics/159.1.303. PMC1461788 11560906

[kiag647-B22] Feil R, Berger F. 2007. Convergent evolution of genomic imprinting in plants and mammals. Trends Genet. 23:192–199. 10.1016/j.tig.2007.02.004. 17316885

[kiag647-B23] Ferguson-Smith AC . 2011. Genomic imprinting: the emergence of an epigenetic paradigm. Nat Rev Genet. 12:565–575. 10.1038/nrg3032. 21765458

[kiag647-B24] Finnegan EJ, Dennis ES. 1993. Isolation and identification by sequence homology of a putative cytosine methyltransferase fromArabidopsis thaliana. Nucleic Acids Res. 21:2383–2388. 10.1093/nar/21.10.2383. PMC309536 8389441

[kiag647-B25] Gehring M et al 2006. DEMETER DNA glycosylase establishes MEDEA polycomb gene self-imprinting by allele-specific demethylation. Cell. 124:495–506. 10.1016/j.cell.2005.12.034. PMC4106368 16469697

[kiag647-B26] Gehring M . 2013. Genomic imprinting: insights from plants. Annu Rev Genet. 47:187–208. 10.1146/annurev-genet-110711-155527. 24016190

[kiag647-B27] Gehring M, Satyaki PR. 2017. Endosperm and imprinting, inextricably linked. Plant Physiol. 173:143–154. 10.1104/pp.16.01353. PMC5210735 27895206

[kiag647-B28] Grini PE et al 1999. Isolation of ethyl methanesulfonate-induced gametophytic mutants in Arabidopsis thaliana by a segregation distortion assay using the multimarker chromosome 1. Genetics. 151:849–863. 10.1093/genetics/151.2.849. PMC1460497 9927475

[kiag647-B29] Grini PE, Jürgens G, Hülskamp M. 2002. Embryo and endosperm development is disrupted in the female gametophytic capulet mutants of Arabidopsis. Genetics. 162:1911–1925. 10.1093/genetics/162.4.1911. PMC1462375 12524359

[kiag647-B30] Guo L et al 2016. The anaphase-promoting complex initiates zygote division in Arabidopsis through degradation of cyclin B1. Plant J. 86:161–174. 10.1111/tpj.13158. 26952278

[kiag647-B31] Gutiérrez-Marcos JF, Costa LM, Evans MMS. 2006. Maternal gametophytic baseless1 is required for development of the central cell and early endosperm patterning in maize (Zea mays). Genetics. 174:317–329. 10.1534/genetics.106.059709. PMC1569813 16849604

[kiag647-B32] Hornslien KS, Miller JR, Grini PE. 2019. Regulation of parent-of-origin allelic expression in the endosperm. Plant Physiol. 180:1498–1519. 10.1104/pp.19.00320. PMC6752901 31064812

[kiag647-B33] Iwase A et al 2017. WIND1 promotes shoot regeneration through transcriptional activation of ENHANCER OF SHOOT REGENERATION1 in Arabidopsis. Plant Cell. 29:54–69. 10.1105/tpc.16.00623. PMC5304349 28011694

[kiag647-B34] Kang I-H, Steffen JG, Portereiko MF, Lloyd A, Drews GN. 2008. The AGL62 MADS domain protein regulates cellularization during endosperm development in Arabidopsis. Plant Cell. 20:635–647. 10.1105/tpc.107.055137. PMC2329934 18334668

[kiag647-B35] Kanno T et al 2008. A structural-maintenance-of-chromosomes hinge domain–containing protein is required for RNA-directed DNA methylation. Nat Genet. 40:670–675. 10.1038/ng.119. 18425128

[kiag647-B36] Kinoshita T et al 2004. One-way control of FWA imprinting in Arabidopsis endosperm by DNA methylation. Science. 303:521–523. 10.1126/science.1089835. 14631047

[kiag647-B37] Köhler C, Grossniklaus U. 2005. Seed development and genomic imprinting in plants. Prog Mol Subcell Biol. 38:237–262. 10.1007/3-540-27310-7_10. 15881898

[kiag647-B38] Köhler C, Page DR, Gagliardini V, Grossniklaus U. 2005. The Arabidopsis thaliana MEDEA Polycomb group protein controls expression of PHERES1 by parental imprinting. Nat Genet. 37:28–30. 10.1038/ng1495. 15619622

[kiag647-B39] Krueger F . 2012. Trim Galore: a wrapper tool around Cutadapt and FastQC to consistently apply quality and adapter trimming to FastQ files, with some extra functionality for MspI-digested RRBS-type (Reduced Representation Bisufite-Seq) libraries. URL http://www.bioinformatics. babraham. ac. uk/projects/trim_galore/. (Date of access: 28/04/2016)

[kiag647-B40] Kwee H-S, Sundaresan V. 2003. The NOMEGA gene required for female gametophyte development encodes the putative APC6/CDC16 component of the Anaphase Promoting Complex in Arabidopsis. Plant J. 36:853–866. 10.1046/j.1365-313X.2003.01925.x. 14675450

[kiag647-B41] Lafon-Placette C, Köhler C. 2014. Embryo and endosperm, partners in seed development. Curr Opin Plant Biol. 17:64–69. 10.1016/j.pbi.2013.11.008. 24507496

[kiag647-B42] Langmead B, Salzberg SL. 2012. Fast gapped-read alignment with Bowtie 2. Nat Methods. 9:357–359. 10.1038/nmeth.1923. PMC3322381 22388286

[kiag647-B43] Li H et al 2009. The sequence alignment/map format and SAMtools. Bioinformatics. 25:2078–2079. 10.1093/bioinformatics/btp352. PMC2723002 19505943

[kiag647-B44] Li H . 2011. Improving SNP discovery by base alignment quality. Bioinformatics. 27:1157–1158. 10.1093/bioinformatics/btr076. PMC3072548 21320865

[kiag647-B45] Lindroth AM et al 2001. Requirement of CHROMOMETHYLASE3 for maintenance of CpXpG methylation. Science. 292:2077–2080. 10.1126/science.1059745. 11349138

[kiag647-B46] Love MI, Huber W, Anders S. 2014. Moderated estimation of fold change and dispersion for RNA-seq data with DESeq2. Genome Biol. 15:550. 10.1186/s13059-014-0550-8. PMC4302049 25516281

[kiag647-B47] Luo A, Shi C, Zhang L, Sun M-X. 2014. The expression and roles of parent-of-origin genes in early embryogenesis of angiosperms. Front Plant Sci. 5:729. 10.3389/fpls.2014.00729. PMC4267172 25566300

[kiag647-B48] Makarevich G, Villar CBR, Erilova A, Köhler C. 2008. Mechanism of PHERES1 imprinting in Arabidopsis. J Cell Sci. 121:906–912. 10.1242/jcs.023077. 18303047

[kiag647-B49] Martienssen RA, Colot V. 2001. DNA methylation and epigenetic inheritance in plants and filamentous fungi. Science. 293:1070–1074. 10.1126/science.293.5532.1070. 11498574

[kiag647-B50] Martin CA, Cogdill KR, Pusey AL, Gehring M. 2026. A transcriptional atlas of early *Arabidopsis* seed development suggests mechanisms for inter-tissue coordination. Nat Plants. 12:1133–1149. 10.1038/s41477-026-02295-8. PMC13197225 42168363

[kiag647-B51] Martin M . 2011. Cutadapt removes adapter sequences from high-throughput sequencing reads. EMBnet J. 17:10–12. 10.14806/ej.17.1.200.

[kiag647-B52] Mathieu O, Reinders J, Caikovski M, Smathajitt C, Paszkowski J. 2007. Transgenerational stability of the Arabidopsis epigenome is coordinated by CG methylation. Cell. 130:851–862. 10.1016/j.cell.2007.07.007. 17803908

[kiag647-B53] Matilla AJ . 2022. Exploring breakthroughs in three traits belonging to seed life. Plants. 11:490. 10.3390/plants11040490. PMC8875957 35214823

[kiag647-B54] Matzke MA, Mosher RA. 2014. RNA-directed DNA methylation: an epigenetic pathway of increasing complexity. Nat Rev Genet. 15:394–408. 10.1038/nrg3683. 24805120

[kiag647-B55] Mike FC and Davis TL. 2022. ggpattern: “ggplot2” Pattern Geoms. https://github.com/coolbutuseless/ggpattern

[kiag647-B56] Montgomery SA, Berger F. 2021. The evolution of imprinting in plants: beyond the seed. Plant Reprod. 34:373–383. 10.1007/s00497-021-00410-7. PMC8566399 33914165

[kiag647-B57] Moreno-Romero J, Del Toro-De León G, Yadav VK, Santos-González J, Köhler C. 2019. Epigenetic signatures associated with imprinted paternally expressed genes in the Arabidopsis endosperm. Genome Biol. 20:41. 10.1186/s13059-019-1652-0. PMC6385439 30791924

[kiag647-B58] Moreno-Romero J, Jiang H, Santos-González J, Köhler C. 2016. Parental epigenetic asymmetry of PRC2-mediated histone modifications in the Arabidopsis endosperm. EMBO J. 35:1298–1311. 10.15252/embj.201593534. PMC4910532 27113256

[kiag647-B59] Nakamura S et al 2010. Gateway binary vectors with the bialaphos resistance gene, bar, as a selection marker for plant transformation. Biosci Biotechnol Biochem. 74:1315–1319. 10.1271/bbb.100184. 20530878

[kiag647-B60] Neff MM, Turk E, Kalishman M. 2002. Web-based primer design for single nucleotide polymorphism analysis. Trends Genet. 18:613–615. 10.1016/S0168-9525(02)02820-2. 12446140

[kiag647-B61] Nowack MK, Ungru A, Bjerkan KN, Grini PE, Schnittger A. 2010. Reproductive cross-talk: seed development in flowering plants. Biochem Soc Trans. 38:604–612. 10.1042/BST0380604. 20298229

[kiag647-B62] Olsen O-A . 2004. Nuclear endosperm development in cereals and Arabidopsis thaliana. Plant Cell. 16(Suppl): S214–S227. 10.1105/tpc.017111. PMC2643391 15010513

[kiag647-B63] Pagnussat GC et al 2005. Genetic and molecular identification of genes required for female gametophyte development and function in Arabidopsis. Development. 132:603–614. 10.1242/dev.01595. 15634699

[kiag647-B64] Pérez-Pérez JM et al 2008. Specialization of CDC27 function in the Arabidopsis thaliana anaphase-promoting complex (APC/C). Plant J. 53:78–89. 10.1111/j.1365-313X.2007.03312.x. 17944809

[kiag647-B65] Phillips AR, Evans MMS. 2011. Analysis of stunter1, a maize mutant with reduced gametophyte size and maternal effects on seed development. Genetics. 187:1085–1097. 10.1534/genetics.110.125286. PMC3070518 21270392

[kiag647-B66] Pignatta D et al 2014. Natural epigenetic polymorphisms lead to intraspecific variation in Arabidopsis gene imprinting. Elife. 3:e03198. 10.7554/eLife.03198. PMC4115658 24994762

[kiag647-B67] Rodrigues JA, Zilberman D. 2015. Evolution and function of genomic imprinting in plants. Genes Dev. 29:2517–2531. 10.1101/gad.269902.115. PMC4699382 26680300

[kiag647-B68] Saleme MdL, Andrade IR, Eloy NB. 2021. The role of anaphase-promoting complex/cyclosome (APC/C) in plant reproduction. Front Plant Sci. 12:642934. 10.3389/fpls.2021.642934. PMC7943633 33719322

[kiag647-B69] Satyaki PRV, Gehring M. 2019. Paternally acting canonical RNA-directed DNA methylation pathway genes sensitize Arabidopsis endosperm to paternal genome dosage. Plant Cell. 31:1563–1578. 10.1105/tpc.19.00047. PMC6635864 31064867

[kiag647-B70] Saze H, Kakutani T. 2007. Heritable epigenetic mutation of a transposon-flanked Arabidopsis gene due to lack of the chromatin-remodeling factor DDM1. EMBO J. 26:3641–3652. 10.1038/sj.emboj.7601788. PMC1949009 17627280

[kiag647-B71] Schindelin J et al 2012. Fiji: an open-source platform for biological-image analysis. Nat Methods. 9:676–682. 10.1038/nmeth.2019. PMC3855844 22743772

[kiag647-B72] Scholl RL, May ST, Ware DH. 2000. Seed and molecular resources for Arabidopsis. Plant Physiol. 124:1477–1480. 10.1104/pp.124.4.1477. PMC1539300 11115863

[kiag647-B73] Schon MA, Nodine MD. 2017. Widespread contamination of Arabidopsis embryo and endosperm transcriptome data sets. Plant Cell. 29:608–617. 10.1105/tpc.16.00845. PMC5435428 28314828

[kiag647-B74] Shirzadi R et al 2011. Genome-wide transcript profiling of endosperm without paternal contribution identifies parent-of-origin–dependent regulation of AGAMOUS-LIKE36. PLoS Genet. 7:e1001303. 10.1371/journal.pgen.1001303. PMC3040660 21379330

[kiag647-B75] St Johnston D, Nüsslein-Volhard C. 1992. The origin of pattern and polarity in the Drosophila embryo. Cell. 68:201–219. 10.1016/0092-8674(92)90466-P. 1733499

[kiag647-B76] Stroud H et al 2014. Non-CG methylation patterns shape the epigenetic landscape in Arabidopsis. Nat Struct Mol Biol. 21:64–72. 10.1038/nsmb.2735. PMC4103798 24336224

[kiag647-B77] Sun H, Schneeberger K. 2015. SHOREmap v3.0: fast and accurate identification of causal mutations from forward genetic screens. Methods Mol Biol. 1284:381–395. 10.1007/978-1-4939-2444-8_19. 25757783

[kiag647-B78] van Ekelenburg YS et al 2023. Spatial and temporal regulation of parent-of-origin allelic expression in the endosperm. Plant Physiol. 191:986–1001. 10.1093/plphys/kiac520. PMC9922421 36437711

[kiag647-B79] Vu TM et al 2013. RNA-directed DNA methylation regulates parental genomic imprinting at several loci in Arabidopsis. Development. 140:2953–2960. 10.1242/dev.092981. PMC3879202 23760956

[kiag647-B80] Wang Y et al 2012. The Arabidopsis APC4 subunit of the anaphase-promoting complex/cyclosome (APC/C) is critical for both female gametogenesis and embryogenesis. Plant J. 69:227–240. 10.1111/j.1365-313X.2011.04785.x. 21910774

[kiag647-B81] Wang Y et al 2013. The Arabidopsis anaphase-promoting complex/cyclosome subunit 1 is critical for both female gametogenesis and embryogenesis(F). J Integr Plant Biol. 55:64–74. 10.1111/jipb.12018. 23206231

[kiag647-B82] Wickham H . 2011. The split-apply-combine strategy for data analysis. J Stat Softw. 40:1–29. 10.18637/jss.v040.i01.

[kiag647-B83] Wickham H . 2016. Ggplot2: elegant graphics for data analysis. Springer-Verlag.

[kiag647-B84] Wolf JB, Wade MJ. 2009. What are maternal effects (and what are they not)? Philos Trans R Soc Lond B Biol Sci. 364:1107–1115. 10.1098/rstb.2008.0238. PMC2666680 19324615

[kiag647-B85] Wolff P et al 2011. High-resolution analysis of parent-of-origin allelic expression in the Arabidopsis Endosperm. PLoS Genet. 7:e1002126. 10.1371/journal.pgen.1002126. PMC3116908 21698132

[kiag647-B86] Xiao W et al 2003. Imprinting of the MEA Polycomb gene is controlled by antagonism between MET1 methyltransferase and DME glycosylase. Dev Cell. 5:891–901. 10.1016/S1534-5807(03)00361-7. 14667411

[kiag647-B87] Yadegari R, Drews GN (2004) Female gametophyte development. Plant Cell 16(Suppl): S133–S141 10.1105/tpc.018192. PMC2643389 15075395

[kiag647-B88] Zhang X, Jacobsen SE. 2006. Genetic analyses of DNA methyltransferases in Arabidopsis thaliana. Cold Spring Harb Symp Quant Biol. 71:439–447. 10.1101/sqb.2006.71.047. 17381326

[kiag647-B89] Zheng B, Chen X, McCormick S. 2011. The anaphase-promoting complex is a dual integrator that regulates both MicroRNA-mediated transcriptional regulation of cyclin B1 and degradation of Cyclin B1 during Arabidopsis male gametophyte development. Plant Cell. 23:1033–1046. 10.1105/tpc.111.083980. PMC3082252 21441434

