# Supplementary material for: Molecular characterization of capulet2 reveals the importance of ANAPHASE PROMOTING COMPLEX 6 maternal expression in endosperm development
Source: Plant Physiol. 2026 Sep 1;202(2):kiag647. doi: 10.1093/plphys/kiag647 (PMC13655448; doi:10.1093/plphys/kiag647)
Supplement: Supplementary file 1 [file kiag647_supplementary_data.zip › SFigures_withledgends_R2.pdf]

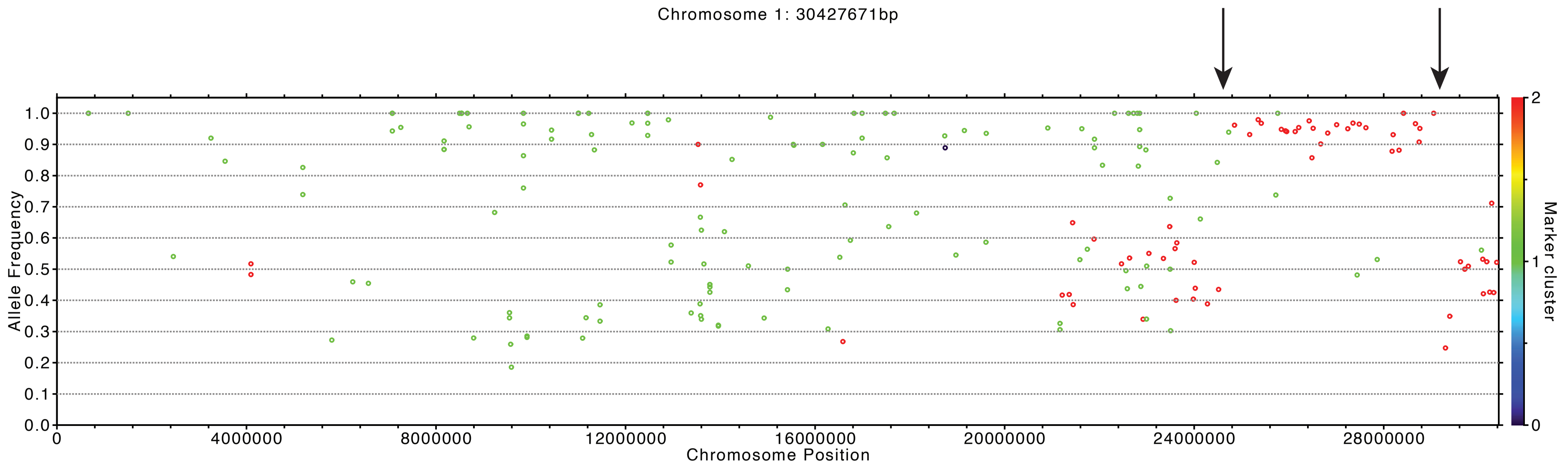

*Points in colors: AF (=alt divided by (alt+ref)) at markers with clustering. alt or ref: coverage of non-reference or reference allele

*rank/cluster table: min_fg_cov max_fg_cov fg_qua bg_mut_cov bg_mut_qua size_cluster

cluster 2: (L:36.9, H:77.1) 220.2 0.0 0.0 63
cluster 1: (L:29.0, H:85.0) 67.9 0.0 0.0 132
cluster 0: (L:48.0, H:66.0) 42.0 40.0 88.0 1

SFigure 1: Distribution of SNPs between *cap2/CAP2* and *CAP2/CAP2*. Allele frequencies across chromosome 1 of putative *cap2* SNPs from comparison 4. Single nucleotide polymorphisms (SNPs) are clustered based on high (red), medium (green) or low (black) quality scores. Black arrows indicate location of recombination breakpoints.

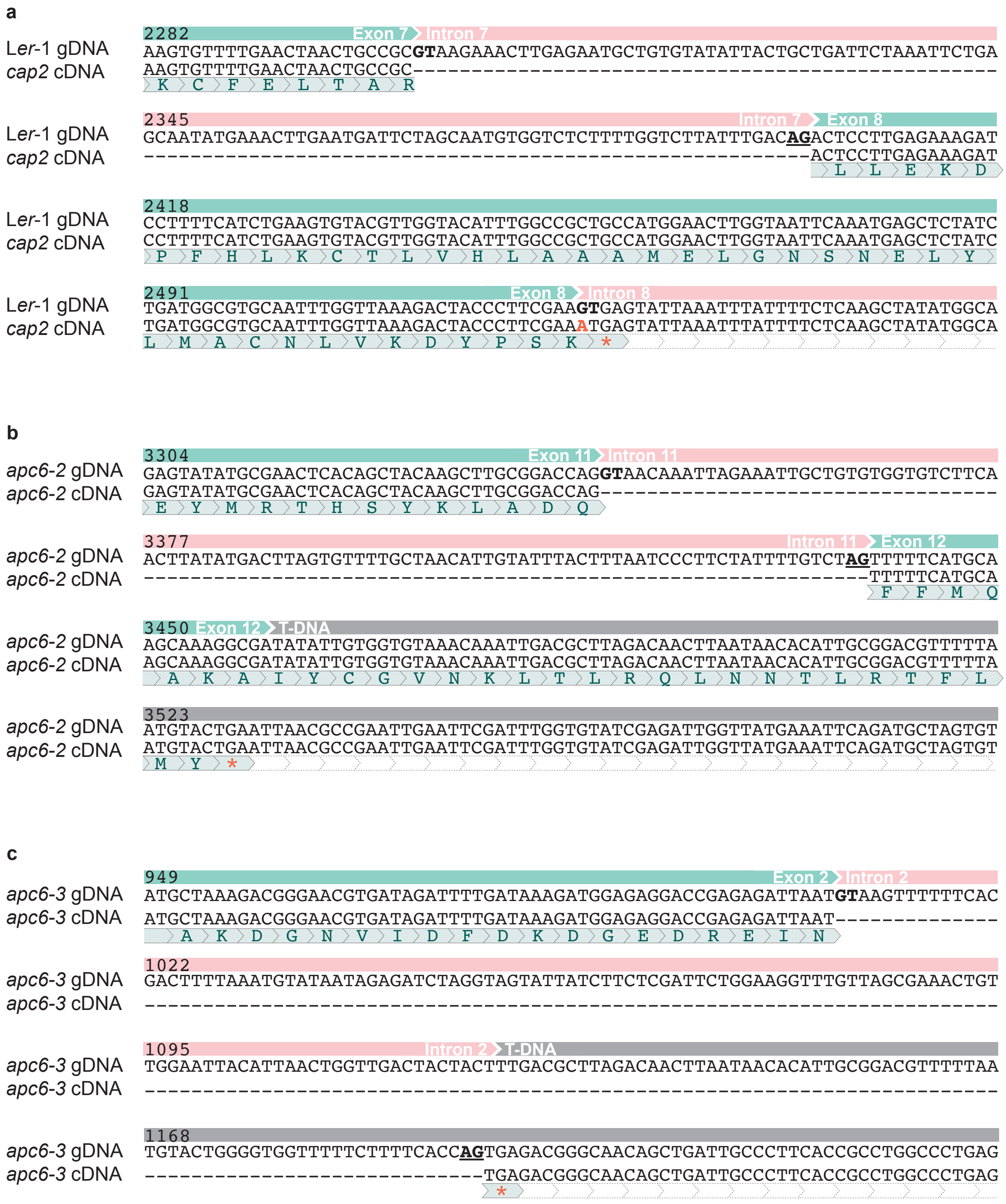

SFigure 2: Sequence alignments of gDNA and *apc6* mutant transcripts. The figure shows alignments between genomic DNA and transcripts from *cap2*, *apc6-2* and *apc6-3* mutant alleles. **a**) Alignment between L*er*-1 gDNA and *cap2* cDNA. The *cap2* mutation (shown in red) disrupts a splice donor site in intron 8, resulting in unspliced transcript with an immediate stop codon. **b**) Alignment between *apc6-2* gDNA and cDNA shows that the T-DNA in exon 12 results in a fusion transcript between exon 12 and T-DNA, leading to an in-frame stop codon. **c**) Alignment between *apc6-3* gDNA and cDNA. Insertion of T-DNA in intron 2 introduces a cryptic splice acceptor site, resulting in a fusion transcript between exon 2 and T-DNA, introducing an immediate stop codon in the transcript. Genomic base count for *APC6* is indicated top left in each sequence line, and exons (green), introns (pink) and T-DNA sequences (grey) are highlighted. Splice donor site sequences and splice acceptor site sequences are marked with bold and bold underlined, respectively. Reading frame and codon translations are indicated for cDNA sequences up till the premature stop codon.

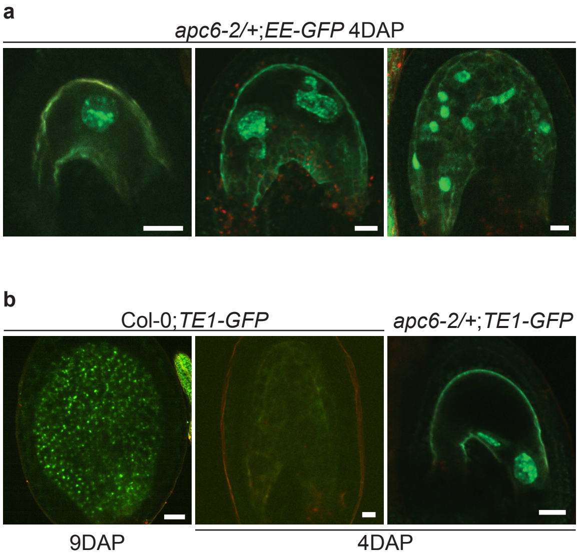

SFigure 3: GFP expression in *apc6-2/+* with endosperm marker lines. *apc6-2/+* mutants were crossed to GFP endosperm marker lines Early Endosperm (*EE-GFP*; a) and Total Endosperm (*TE1-GFP*; b) and propagated to obtain homozygous marker lines. Selfed *apc6-2/+;EE-GFP* and *apc6-2/+;TE1-GFP* were inspected at 4 days after pollination (DAP) using confocal microscopy. All seeds were counter-stained with Propidium Iodide (PI; red). a) *apc6-2/+;EE-GFP* seeds displaying *apc6-2* endosperm phenotypes with one, two and more than eight enlarged nuclei (left to right). GFP signal was only observed in fertilized seeds, not in unfertilized ovules (n=147 ovules). Scale bar = 20 µm. b) The *TE1-GFP* marker is expressed at 9 DAP, but not at 4 DAP. In 4 DAP *apc6-2/+;TE1-GFP*, a seed expressing GFP was only observed once (n = 14 siliques). No GFP signal was observed in *apc6-2/+;TE1-GFP* seeds at 5DAP and 6DAP. Scale bar = 50 µm (9 DAP) and 20 µm (4 DAP).

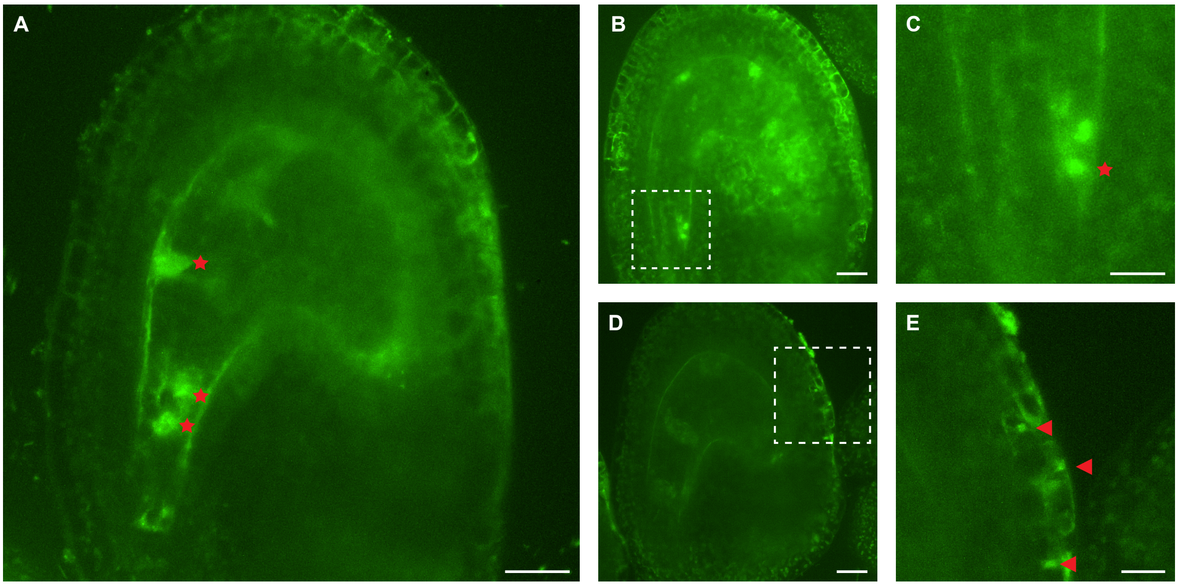

SFigure 4: Verification of the *APC6* transgene functionality using confocal microscopy. 1 day after pollination (DAP) (a, d, e) and 2 DAP (b, c) seeds from L*er*-1 transformed with *proAPC6:APC6-GFP*. a) GFP signal is observed in endosperm nuclei. b,c) GFP signal is observed in endosperm nuclei, but not in the embryo. d,e) Weak GFP signal is detected in seed coat nuclei. Asterisks indicate endosperm nuclei; Arrowheads indicate seed coat nuclei. Scale bar = 20 µm a, b, d; 10 µm c, e.

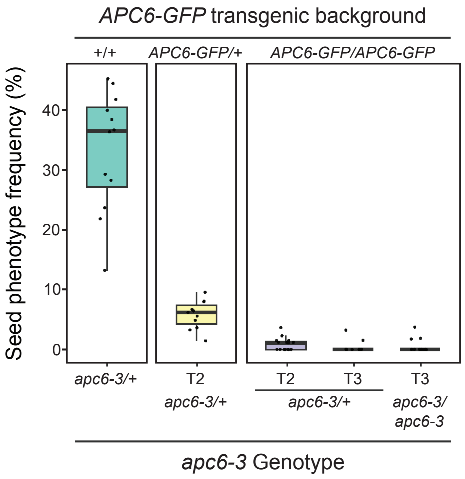

SFigure 5: An *APC6* transgene can complement the *apc6-3/+* mutant phenotype. In the T2 generation, the seed phenotype frequency of *apc6-3/+* is substantially reduced in a hemizygous transgenic background and fully alleviated in a homozygous transgenic background. Two independent T2 *apc6-3/+* lines in a homozygous transgenic background, were propagated to the T3 generation and full rescue of the seed phenotype is observed in *apc6-3/+* and homozygous *apc6-3/apc6-3*.

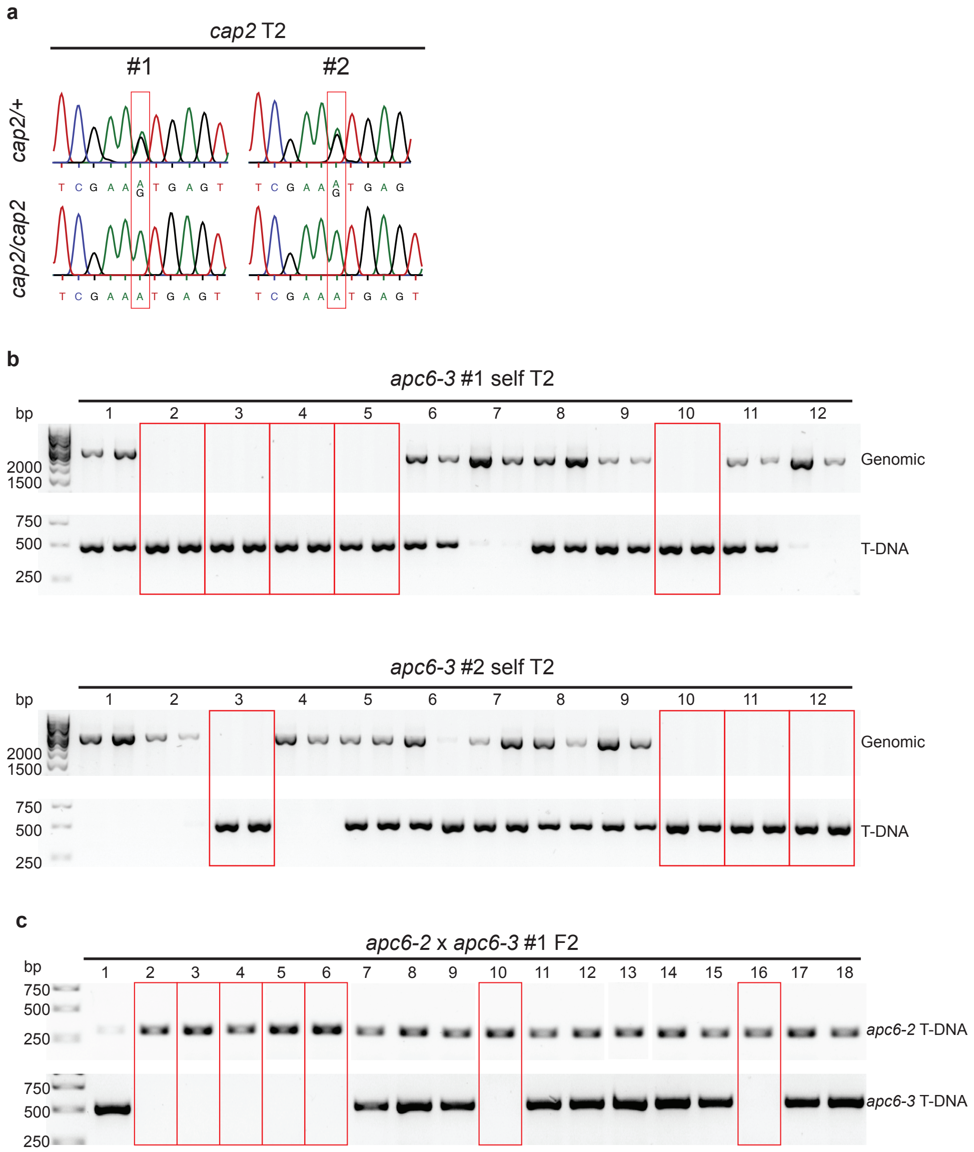

SFigure 6: Identification of homozygous *cap2*, *apc6-3* and *apc6-2* plants after complementation with an *APC6* transgene. a) Two introgressed *cap2* lines (3x into Col-0 (#1) and 3x into L*er*-1 (#2)) were transformed with the transgene and propagated to the T2 generation and heterozygous (*cap2/+*) and homozygous (*cap2/cap2*) individuals were identified. Red squares indicate location of the *cap2* SNP. b) Two independent T2 *apc6-3/+* lines in a homozygous transgenic background (*apc6-3* #1 and *apc6-3* #2), were propagated to the T3 generation and individuals were genotyped in duplicate using primers specific to the genomic *APC6* locus, independently of the transgene. Red squares indicate homozygous *apc6-3* individuals. c) Homozygous *apc6-3* individuals from line #1 were crossed to *apc6-2/+* plants and offspring were propagated to the F2 generation. F2 offspring were first genotyped using primers specific to the *apc6-2* T-DNA, then with primers specific to the *apc6-3* T-DNA. Both homozygous *apc6-2* (red square) and transhomozygous *apc6-2/apc6-3* individuals were identified.

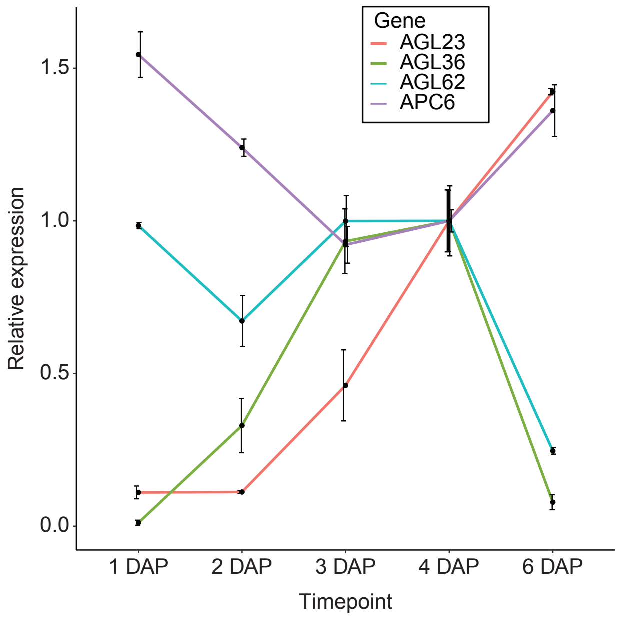

SFigure 7: Relative expression of *APC6* throughout seed development. Expression of *APC6*, *AGL36*, *AGL23* and *AGL62* at different timepoints (1, 2, 3, 4 and 6 days after pollination (DAP)). Expression levels of two biological replicates were calculated relative to the expression level at 4 DAP and the mean was plotted with standard deviation.

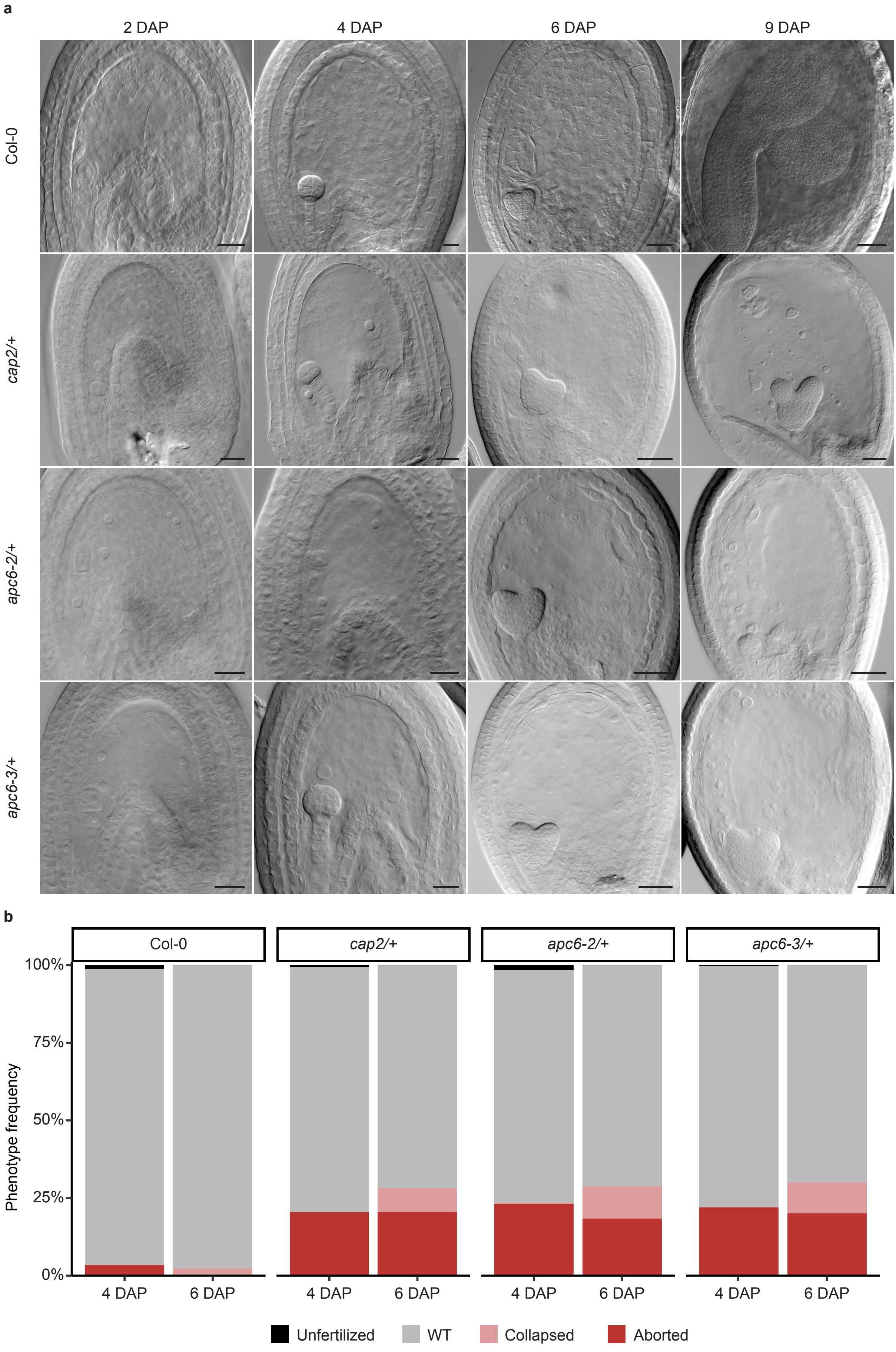
a
2 DAP
4 DAP
6 DAP
9 DAP
Col-0
cap2/+
apc6-2/+
apc6-3/+
b
Col-0
cap2/+
apc6-2/+
apc6-3/+
100%
75%
50%
25%
0%
Phenotype frequency
4 DAP
6 DAP
Unfertilized
WT
Collapsed
Aborted

SFigure 8: Seed development in *APC6* mutants and wild-type. a) Seed developmental phenotypes for *cap2/+*, *apc6-2/+* and *apc6-3/+* crossed maternally to wild-type Col-0 compared to selfed Col-0 wild-type. At 2 and 4 days after pollination (DAP), few and enlarged endosperm nuclei can be observed whereas at 6 and 9 DAP, the endosperm has not undergone cellularization. Embryo development and seed size seem normal but delayed for *APC6* mutants at 6 and 9 DAP. Scale bar = 20 µm 2 and 4 DAP; 50 µm 6 and 9 DAP. **b)** Classification of *cap2/+*, *apc6-2/+* and *apc6-3/+* seed phenotypes at 4 and 6 DAP. Collapsed seeds were indistinguishable from unfertilized or aborted seeds.

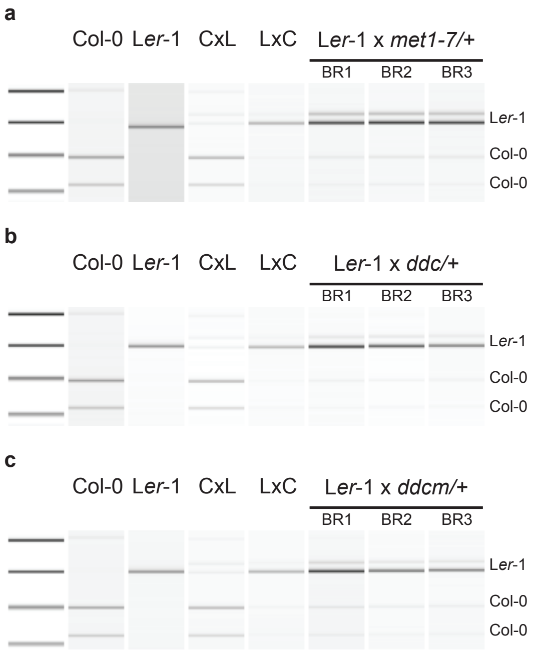

d

Cross	m:p ratio
L*er*-1 x *met1-7/+*	9.1 ± 1.0
L*er*-1 x *ddc/+*	9.8 ± 2.1
L*er*-1 x *ddcm/+*	7.9 ± 0.5

SFigure 9: Imprinting of *APC6* is not regulated by canonical DNA methylation. Single nucleotide polymorphism (SNP) digestion analysis of 4 days after pollination (DAP) seeds from crosses of L*er*-1 with DNA methyltransferase mutants (in a Col-0 background). a-c) One replicate of homozygous wild-type controls is included as accession-specific cDNA, as well as one replicate of reciprocal crosses between wild-type Col-0 and L*er*-1. One of two bioanalyzer replicates is presented with three biological replicates. In all crosses of L*er*-1 with hemizygous *met1-7/+* (a), hemizygous *ddc/+* (b) or hemizygous *ddcm/+* (c), *APC6* is maternally expressed and no reactivation of the paternal allele is observed. d) Molarity ratios of maternal and paternal digested fragments from bioanalyzer profiles (a-c). Parental ratios are determined from all biological replicates and both bioanalyzer technical replicates; sd, standard deviation.

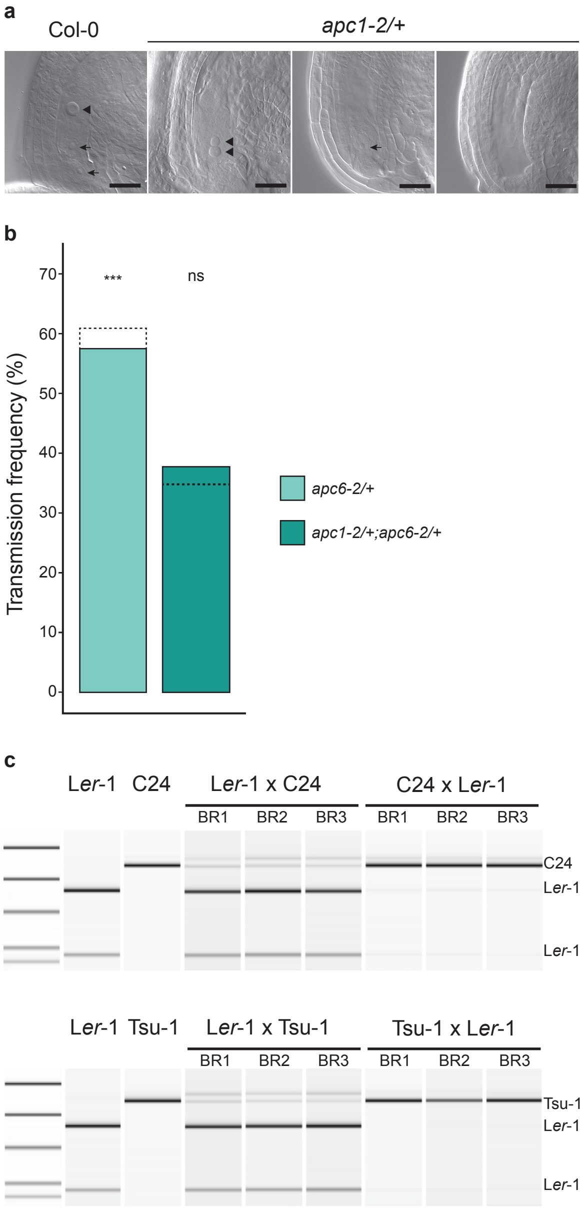

SFigure 10: Characterization of *apc1-2* ovules, *apc1-2/+;apc6-2* mutant allele transmission and maternal expression of *APC1*. a) Ovules of *apc1-2/+* at 3 days after emasculation (DAE) show developmental abnormalities and often lack one or more cells or nuclei in the micropylar embryo sac compared to Col-0, where the central cell, egg cell and both synergids are present (only one synergid is visible in this figure). b) Transmission frequency of the *apc6-2* allele (n = 2085 seedlings) and the *apc1-2* and *apc6-2* alleles together (n = 303 seedlings) in offspring resulting from selfed *apc1-2/+;apc6-2/+* double mutants compared to expected frequencies (dashed line). In an *apc1-2/+;apc6-2/+* double mutant background, transmission of the *apc6-2* allele is significantly reduced. Statistics were performed according to the Chi square test; *** p-value < 0.001. c) Imprinting analysis of *APC1* using accession-specific restriction digestion of SNPs in reciprocal crosses between L*er*-1, and C24 and Tsu-1. cDNA was generated from 4 days after pollination (DAP) crossed seeds. One replicate of homozygous wild-type controls is included as accession-specific cDNA reference and three biological replicates for each reciprocal cross are shown. One of two bioanalyzer replicates is presented and the accession of the maternal contributor is named first. *APC1* is maternally expressed in all crosses, in both directions.
