# Supplementary material for: Molecular characterization of capulet2 reveals the importance of ANAPHASE PROMOTING COMPLEX 6 maternal expression in endosperm development
Source: Plant Physiol. 2026 Sep 1;202(2):kiag647. doi: 10.1093/plphys/kiag647 (PMC13655448; doi:10.1093/plphys/kiag647)
Supplement: Supplementary file 1 [file kiag647_supplementary_data.zip › STable 1 SNP identification_update_v1.pdf]

STable 1: Identification of the candidate SNPs for *capulet2*. a) Twelve *cap2/CAP2* individuals were compared with twelve *CAP2/CAP2* individuals and SNPs between the *ADH1* molecular marker and *gl2* visible marker were identified. The candidate SNPs were subjected to several filters and thresholds. b) The remaining three candidates were identified across all comparisons and localized to the *ANAPHASE PROMOTING COMPLEX SUBUNIT 6* (*APC6*), *STRUBBELIG-RECEPTOR FAMILY 5* (*SRF5*) and in an intergenic region. 1 Minimum BAQ score (Li, 2011) of 70. 2 EMS mutagenesis results in a G > A conversion. 3 Allele frequency between 0.25 and 0.75.

a

Comparison[1]	Total SNPs	≥70 BAQ[2]	G > A[3]	Allele frequency[4]
1	167	24	10	7
2	129	18	9	8
3	150	24	11	9
4	173	13	9	7
5	145	11	9	4
6	152	13	8	5
7	110	16	12	8
8	144	16	11	7
9	133	12	5	4
10	128	18	9	5
11	112	11	8	8
12	108	14	8	8

b

Location	Type of mutation	AGI	Gene
29,619,446	Splice donor site change	AT1G78770	*ANAPHASE PROMOTER COMPLEX SUBUNIT 6* (*APC6*)
29,708,804	CDS; nonsynonymous	AT1G78980	*STRUBBELIG-RECEPTOR FAMILY 5* (*SRF5*)
29,783,917	Intergenic	-	-
