# Supplementary material for: Molecular characterization of capulet2 reveals the importance of ANAPHASE PROMOTING COMPLEX 6 maternal expression in endosperm development
Source: Plant Physiol. 2026 Sep 1;202(2):kiag647. doi: 10.1093/plphys/kiag647 (PMC13655448; doi:10.1093/plphys/kiag647)
Supplement: Supplementary file 1 [file kiag647_supplementary_data.zip › STable 2 Embryo sac counting.pdf]

STable 2: Phenotypic characterization of the embryo sac. Mature ovules from wild-type Col-0, *cap2/+*, *apc6-2/+* and *apc6-3/+* were manually dissected at 3 days after emasculation (DAE) and the embryo sac was characterized as mature, non-developed or not observable (NA).

Plant	Mature embryo sac	NA	Non-developed	Total
Col-0	81%	17%	2%	185
cap2/+	81%	17%	2%	161
apc6-2/+	84%	16%	-	97
apc6-3/+	77%	20%	3%	187
