# Supplementary material for: Molecular characterization of capulet2 reveals the importance of ANAPHASE PROMOTING COMPLEX 6 maternal expression in endosperm development
Source: Plant Physiol. 2026 Sep 1;202(2):kiag647. doi: 10.1093/plphys/kiag647 (PMC13655448; doi:10.1093/plphys/kiag647)
Supplement: Supplementary file 1 [file kiag647_supplementary_data.zip › STable 3 Primers _update_v3.pdf]

STable 3: Primer name, sequence and comments.

Primer	Oligo sequence (5' –> 3')	Comments
LBa1	TGGTTCACGTAGTGGGCCATCG	Genotyping Salk T-DNA left border for *apc6-3/drm1;drm2/apc1-2;* sequencing *apc6-3* mutant transcripts
LBb1.3	ATTTTGCCGATTTCGGAAC	Genotyping Salk T-DNA left border for *met1-7/cmt3-11*
LB3	TAGCATCTGAATTTCATAACCAATCTCGATACAC	Genotyping Sail T-DNA left border for *apc6-2;* sequencing *apc6-2* mutant transcripts
APC6_SAIL442_F11_Fp	TGCCAACTCTGTACATTGGAATGGAG	Genotyping genomic forward for *apc6-2*; Sail T-DNA left/right border for *apc6-2*
APC6_SAIL442_F11_Rp	GACCACAGTTGGTTCCCAGG	Genotyping genomic reverse for *apc6-2*
APC6_SLK008789Nw_Fp	TGATGCTAAAGACGGGAACGTGA	Genotyping genomic forward for *apc6-3*; Salk T-DNA left border for *apc6-3*
APC6_SALK_008789_Rp	AGCACAGAGGATCAGCCTTGA	Genotyping genomic reverse for *apc6-3*
APC6_intron1-3_Rp	CACAGAGGATCAGCCTTGATAGCA	Genotyping Salk T-DNA right border *apc6-3*
APC6_intron7-9_Fp	GTGGTAGTGTCTCTGGCTCATCT	Genotyping forward for *capulet2;* sequencing *cap2* mutant transcripts
APC6_3DOWN_2_Rp	GTAACAGTCTCTTGGTTATTGATGCATGGA	Genotyping reverse for *capulet2*
DRM2_SP	AGTTAGCCCGAGGGCCACCTTT	Genotyping genomic *drm1;drm2*
DRM2_ASP	CGCCTGCCAGATTGTTACAAGG	Genotyping genomic *drm1;drm2*; Salk T-DNA left border for *drm1;drm2*
cap2_SNP_Rp	GGTTAACTATCCTAGCTAGGATTGGGA	Genotyping reverse for *capulet2;* sequencing *cap2* mutant transcripts
cmt3_SALK_148381_ASP	CGTGAAGGTTGGCATATTCTTCTG	Genotyping genomic *cmt3-11*; Salk T-DNA left border for *cmt3-11*
cmt3_SALK_148381_SP	AGCTATGTCGACAGGGTTGTGC	Genotyping genomic *cmt3-11*
met1_SALK076522_Rp	CAGAACAGGTTTCCACCAAGG	Genotyping genomic *met1-7*; Salk T-DNA left border for *met1-7*
met1_SALK076522_Lp	TCAATGAGCAGTGGAAGCAAG	Genotyping genomic *met1-7*
APC6_ecot_SNP1_Fp	GCTGAGTACTACCATCAATG	*APC6 Ler*-1 x Col-0/Tsu-1/C24 SNP digestion forward primer
APC6_ecot_SNP1_Rp	CACGCCATTAGATAGAGC	*APC6 Ler*-1 x Col-0/Tsu-1/C24 SNP digestion reverse primer
ColC24_Fp	CTTGGTTTGCGGTGGGTTG	*APC6* Col-0 x C24 SNP digestion forward primer
ColC24_BglII_Rp	CGAGCTGGTGAGAATGAGAGATC	*APC6* Col-0 x C24 SNP digestion reverse primer
APC6-F	TGGCGCGCCCGAGGTATGGATAAAACTCTATCTCCGT	Amplifying *APC6* with promoter for construction of *proAPC6:APC6:GFP*
APC6-R	AATCCCGGGGCAGAGCTCAACCTTTGAATCAACCCCG	Amplifying *APC6* with promoter for construction of *proAPC6:APC6:GFP*
apc1-2_Fp	CTTGGTCGAGGGACAGAAGC	Genotyping genomic forward for *apc1-2*
apc1-2_Rp	CAGGTTCCTCTAGTGGATCTCTTAGAATCAG	Genotyping genomic reverse for *apc1-2;* Salk T-DNA
